# Precise Control of Intracellular Trafficking and Receptor‐Mediated Endocytosis in Living Cells and Behaving Animals

**DOI:** 10.1002/advs.202405568

**Published:** 2024-10-14

**Authors:** Shiau‐Chi Chen, Neng‐Jie Zeng, Grace Y. Liu, Hsien‐Chu Wang, Tzu‐Ying Lin, Yi‐Ling Tai, Chiao‐Yun Chen, Yin Fang, Yi‐Chien Chuang, Ching‐Lin Kao, Hsuan Cheng, Bing‐Huang Wu, Pin‐Chiao Sun, Odvogmed Bayansan, Yu‐Ting Chiu, Chi‐Hsuan Shih, Wen‐Hong Chung, Jia‐Bin Yang, Lily Hui‐Ching Wang, Po‐Han Chiang, Chun‐Hao Chen, Oliver I. Wagner, Yi‐Ching Wang, Yu‐Chun Lin

**Affiliations:** ^1^ Institute of Molecular Medicine National Tsing Hua University Hsinchu 300044 Taiwan; ^2^ Institute of Molecular and Cellular Biology National Tsing Hua University Hsinchu 300044 Taiwan; ^3^ Institute of Molecular and Cellular Biology National Taiwan University Taipei 106319 Taiwan; ^4^ School of Medicine National Tsing Hua University HsinChu 300044 Taiwan; ^5^ Department of Medical Science National Tsing Hua University Hsinchu 300044 Taiwan; ^6^ Institute of Biomedical Engineering National Yang Ming Chiao Tung University Hsinchu 300093 Taiwan; ^7^ Department of Pharmacology College of Medicine National Cheng Kung University Tainan 701401 Taiwan

**Keywords:** chemical‐inducible dimerization, cytoskeleton, optogenetics, receptor‐mediated endocytosis, vesicular trafficking, virus entry

## Abstract

Intracellular trafficking, an extremely complex network, dynamically orchestrates nearly all cellular activities. A versatile method that enables the manipulation of target transport pathways with high spatiotemporal accuracy in vitro and in vivo is required to study how this network coordinates its functions. Here, a new method called RIVET (Rapid Immobilization of target Vesicles on Engaged Tracks) is presented. Utilizing inducible dimerization between target vesicles and selective cytoskeletons, RIVET can spatiotemporally halt numerous intracellular trafficking pathways within seconds in a reversible manner. Its highly specific perturbations allow for the real‐time dissection of the dynamic relationships among different trafficking pathways. Moreover, RIVET is capable of inhibiting receptor‐mediated endocytosis. This versatile system can be applied from the cellular level to whole organisms. RIVET opens up new avenues for studying intracellular trafficking under various physiological and pathological conditions and offers potential strategies for treating trafficking‐related disorders.

## Graphical abstract

The RIVET (Rapid Immobilization of Target Vesicles on Engaged Tracks) system enables spatiotemporal perturbation of specific intracellular trafficking pathways and receptor‐mediated endocytosis in living cells and organisms. This is accomplished through chemically or light‐inducible dimerization between target vesicles, granules, or receptors and their associated railways, such as microtubules and actin filaments. This powerful tool allows for the dissection of dynamic intracellular trafficking processes and holds potential therapeutic applications, such as blocking viral entry.

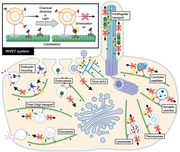

## 1 Introduction

Intracellular trafficking is a fundamental and conserved mechanism present in all eukaryotic cells, facilitating the transport of signaling molecules and cargoes essential for proper cell activities.^[^ ^1^, ^2^, ^3^, ^4^, ^5^, ^6^ ^]^ Cells boast an intricately complex transport network that meticulously regulates intracellular transport both spatially and temporally.^[^ ^7^, ^8^, ^9^ ^]^ This network is under the tight regulation of various protein families, including Rab small GTPases, SNARE proteins, Golgins, different coat proteins, and others.^[^ ^1^, ^7^, ^8^, ^9^ ^]^ Unsurprisingly, defects in these control systems lead to a broad spectrum of human diseases.^[^ ^4^, ^5^, ^10^, ^11^, ^12^ ^]^

To unravel the molecular intricacies of intracellular transport, tools that enable precise perturbation of specific transport pathways are imperative. Over the past several decades, numerous chemical reagents for blocking intracellular transport pathways have been widely used.^[^ ^13^, ^14^, ^15^ ^]^ However, these chemicals often target multiple regulators and pathways, resulting in off‐target effects.^[^ ^16^, ^17^ ^]^ Additionally, the gene depletion of endogenous regulators or overexpression of dominant negative mutants is a common strategy to inhibit specific pathways. However, the slow perturbation process of these methods, typically taking several days, makes it challenging to dissect the dynamic processes of vesicular trafficking.

Few methods, such as RUSH (Retention using selective hooks), have been designed to temporally control specific vesicular transport.^[^ ^18^ ^]^ However, this method is primarily applicable to secretory protein traffic, and its off rate is relatively slow. Moreover, biotin, a ligand used to initiate RUSH, is present in the daily diet of several species, including Drosophila, leading to a high leakage background in those systems.^[^ ^19^, ^20^ ^]^ Light‐induced vesicle clustering has also been utilized to block the function of Rab vesicles and synaptic vesicles on the timescale of minutes.^[^ ^21^, ^22^, ^23^ ^]^ Furthermore, intracellular components can be mislocalized to the minus or plus end of microtubule networks within minutes by linking them with dynein or kinesin motors, respectively.^[^ ^24^, ^25^, ^26^ ^]^ Although these studies have demonstrated the potential for spatiotemporally controlling the positioning of target vesicles, they still require minutes to exert their effects. Thus far, a versatile tool that can precisely perturb a wide spectrum of intracellular trafficking pathways with high spatiotemporal accuracy is still lacking.

We have developed a new tool named RIVET (Rapid Immobilization of target Vesicles on Engaged Tracks) based on established dimerization systems.^[^ ^27^, ^28^ ^]^ RIVET enables the swift perturbation of target vesicles or granules, along with their cargo, on selective cytoskeletons within seconds. This powerful tool has allowed us to uncover how synaptic vesicle transport on microtubules regulates animal behavior. Additionally, the specific inhibition of receptor‐mediated endocytosis by RIVET proves effective in attenuating SARS‐CoV‐2 virus entry. RIVET holds the potential to unveil new insights into intracellular trafficking pathways and offers promising clinical applications.

## 2 Results

### 2.1 Design of a Versatile Tool for Rapidly Inhibiting Target Intracellular Trafficking

**Figure 1:**
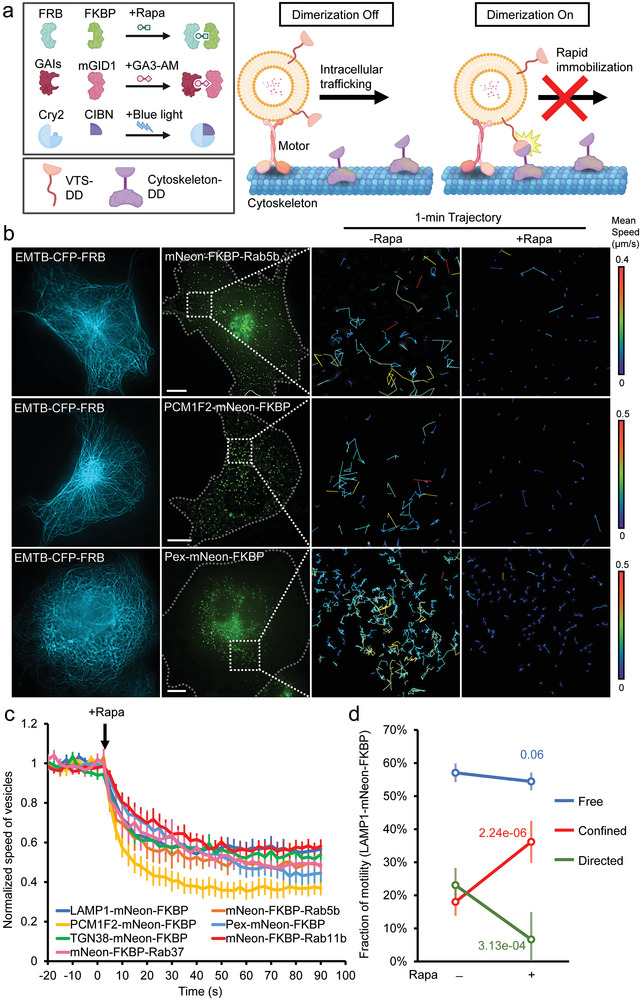
RIVET is a versatile tool to rapidly immobilize intracellular trafficking. a) Schematic representation of the inducible vesicle immobilization system. Dimerization of three different pairs, FRB/FKBP, GAIs/mGID1, and Cry2/CIBN, can be triggered by rapamycin (Rapa), GA3‐AM, and blue light, respectively. One of the dimerizing domains (DDs) is fused with different vesicle‐targeting sequences (VTSs), while the other is fused with cytoskeleton‐associated proteins. Dimerization upon certain stimuli (Rapa, GA3‐AM, or light illumination) traps and immobilizes target vesicles onto DD‐labeled cytoskeletons. The graph was created using BioRender. b) COS7 cells co‐transfected with EMTB‐CFP‐FRB (blue) and indicated mNeon‐FKBP tagged vesicular markers (green) were treated with rapamycin (Rapa, 100 nM) for the inducible vesicle immobilization. The enlarged mean speed trajectories of indicated vesicular markers are shown. Scale bars, 10 µm. c) The normalized mean speed of the indicated vesicles upon rapamycin treatment is shown. *n* = 10, 9, 10, 9, 10, 9, and 8 cells for LAMP1‐mNeon‐FKBP, mNeon‐FKBP‐Rab5b, PCM1F2‐mNeon‐FKBP, Pex‐mNeon‐FKBP, TGN38‐mNeon‐FKBP, mNeon‐FKBP‐Rab11b, and mNeon‐FKBP‐Rab37 groups, respectively, from 3 to 4 independent experiments. Data are shown as mean ± SEM. d) The motility type of LAMP1 changed after 10 min of rapa treatment using RIVET. The red, green, and blue lines represent the percentage of LAMP1‐vesicles with confined behavior, directed movement, and free diffusion, respectively. Data are shown as mean ± SD. *n* = 9 cells from 3 independent experiments. Student's *t*‐tests were performed, with the resulting *p*‐values indicated.

We here aim to develop a versatile tool for manipulating various intracellular trafficking pathways, and therefore designed our system based on a conserved mechanism of trafficking, as vesicles or granules move along cytoskeletons. We tested the possibility of tethering target vesicles to their railways using inducible dimerization systems. Rapamycin inducible dimerization between FKBP (FK506‐binding protein) and FRB (FKBP‐rapamycin binding domain), a widely used chemically inducible dimerization (CID) system,^[^ ^27^, ^29^ ^]^ was employed to prove the concept. One of the binding partners, FKBP, and a yellow fluorescent protein, mNeon, were fused with targeting proteins of the vesicle of interest (VOI). The other binding partner, FRB, and a cyan fluorescent protein, CFP, were attached to microtubules (MTs) by conjugating with an MT‐binding protein, EMTB (the MT‐binding domain of ensconsin; **Figure** **1**a).^[^ ^30^, ^31^ ^]^ Expression of EMTB‐CFP‐FRB in COS7 cells did not affect vesicular dynamics, as the comparable velocities of LAMP1‐mNeon‐FKBP labeled lysosomes, PCM1F2‐mNeon‐FKBP‐labeled centriolar satellites,^[^ ^31^ ^]^ and TGN38‐mNeon‐FKBP‐labeled post‐Golgi vesicles were observed in the presence and absence of EMTB‐FRB expression (Figure S1a–f, Supporting Information). Moreover, FKBP tagging on VOIs did not affect their motility as evidenced by the similar velocities of VOIs with and without FKBP tagging (Figure S1g,h and Video S1, Supporting Information). Fluorescence Recovery After Photobleaching (FRAP) showed that 62.9 ± 14.2% of EMTB are immobilized, suggesting its potential use for trapping vesicles (Figure S2 and Video S2, Supporting Information). We next tested whether dimerization between VOIs and MTs can be triggered by the CID system. Addition of rapamycin rapidly trapped cytosolic mNeon‐FKBP onto EMTB‐CFP‐FRB‐labeled MTs, accompanied by an increased FRET (fluorescence resonance energy transfer) signal derived from proximity between mNeon and CFP (Half time *t*_1/2_ = 4.9 ± 1.3 s). Dimerization between LAMP1‐mNeon‐FKBP‐labeled lysosomes and MTs could also be triggered in cellulo with a fast induction rate (*t*_1/2_ = 11.4 ± 1.9 s; Figure S3 and Video S3, Supporting Information). Such dimerization between VOIs on MTs also dramatically slowed down the motility of lysosomes (*t*_1/2_ = 11.0 ± 3.1 s; Figure S4 and Video S4, Supporting Information). In addition to lysosomes, by tagging FKBP to different markers of VOIs, the CID system swiftly halted different trafficking pathways, including endosomes (labeled by mNeon‐FKBP‐Rab5b; *t*_1/2_ = 11.8 ± 4.0 s), centriolar satellites (labeled by PCM1F2‐mNeon‐FKBP; *t*_1/2_ = 10.5 ± 2.5 s),^[^ ^31^ ^]^ peroxisomes (labeled by Pex‐mNeon‐FKBP; *t*_1/2_ = 20.3 ± 5.0 s), post‐Golgi vesicles (labeled by TGN38‐mNeon‐FKBP; *t*_1/2_ = 9.0 ± 1.3 s), recycling endosomes (labeled by mNeon‐FKBP‐Rab11b; *t*_1/2_ = 12.5 ± 2.8 s), and exocytic vesicles (labeled by mNeon‐FKBP‐Rab37; *t*_1/2_ = 12.3 ± 3.6 s; Figure 1b,c; Table S1, Figure S4 and Video S4, Supporting Information). Vesicle motility cannot be perturbed in the absence of either chemical dimerizers or dimerizing domains tagged on VOIs (Figure S5 and Video S5, Supporting Information). We next evaluated whether the immobilization of marker proteins could efficiently slow down their labeled components. Lysosomes were labeled by LysoTracker dye in COS7 cells expressing EMTB‐CFP‐FRB and LAMP1‐mNeon‐FKBP. The addition of rapamycin halted the LAMP1‐positive components and simultaneously triggered lysosome immobilization (Figure S6 and Video S6, Supporting Information), confirming that our system halts both signature proteins and their target components. Moreover, due to the irreversibility of CID, a transient rapamycin treatment (5 min) is sufficient to maintain vesicle immobilization for at least 24 h (Figure S7 and Video S7, Supporting Information). Besides EMTB, we also tested the possibility of using other MAPs. Specifically, we tested MAPTau (Microtubule‐associated protein Tau) and found MAPTau associated with different MT subtypes compared to EMTB (Figure S8a,b, Supporting information). MAPTau was tagged with FRB, allowing the resulting protein to rapidly stall VOIs through a dimerization reaction (*t*_1/2_ = 17.0 ± 2.2 s; Figure S8c,d and Video S8, Supporting Information). According to the design and features, we named this tool RIVET (Rapid Immobilization of target Vesicles on Engaged Tracks). In summary, these data show that RIVET enables rapid immobilization of target VOIs on their railways on the timescale of seconds, and it can be generally applied to various trafficking pathways.

### 2.2 RIVET Perturbs Specific Cytoskeleton‐Dependent Vesicular Trafficking

It is noteworthy that RIVET only reduced the original velocity by 44.4–63.5% in different intracellular trafficking pathways (Figure 1c; Table S1, Supporting Information). There are several possibilities to explain this incomplete vesicle immobilization. 1) The affinity of FRB/FKBP dimerization may not be strong enough to fully halt vesicle motility. To assess this, we enhanced dimerization by attaching tandem FRB (EMTB‐CFP‐FRBx2) onto MTs. The comparison between EMTB‐FRB and EMTB‐FRBx2 groups showed no difference in their velocity reduction of lysosomes upon dimerization reaction (Figure S9 and Video S9, Supporting Information), suggesting that one copy of FRB/FKBP dimerization is enough for vesicle immobilization on MTs. 2) The existence of MT‐independent vesicular movement. To address this, we measured the motility of LAMP1‐labeled lysosomes after nocodazole‐induced MT disassembly (Figure S10a, Supporting Information). Nocodazole treatment only reduced 35.8 ± 1.9% and 48.2 ± 2.1% of LAMP1‐ and TGN38‐labeled vesicle motility, respectively (Figure S10b,c,e,f and Video S10, Supporting Information). Analysis of motility type confirmed that nocodazole treatment converted the directed movement of these vesicles (considered as movement along cytoskeletons) to a confined signal.^[^ ^32^ ^]^ ≈58.1% and 60.3% of LAMP1‐ and TGN38‐vesicles move with random directions, respectively (defined as “Free”), which were not affected by nocodazole treatments, implying that these populations of vesicles move via MT‐independent mechanisms (Figure S10d,g, Supporting Information). In agreement with this assumption, tethering LAMP1‐positive lysosomes on MTs converted their directed movement to confined without affecting 57.0% of MT‐independent diffused motility (Figure 1d). All these results demonstrate that the dimerization reaction efficiently inhibits MT‐dependent motility of intracellular components. To test whether actin filaments are involved in the MT‐independent motility we proposed, we attempted to trap VOIs on actin filaments following nocodazole‐induced MT disassembly. In this experiment, LifeAct, an actin filament‐binding peptide, was tagged with FRB. The local and random movement of TGN38‐mNeon‐FKBP vesicles caused by MT disassembly was further suppressed by trapping them on LifeAct‐FRB‐labeled actin filaments (Figure S11 and Video S11, Supporting Information). This result indicates that actin filaments are at least partially involved in vesicle motility in an MT‐independent manner.

### 2.3 RIVET can be Triggered by Various Dimerization Systems in Different Cell Types

We investigated whether RIVET could be triggered by a rapamycin‐independent CID system. To test this, we employed a plant hormone, gibberellin, which mediated the CID system to manipulate vesicular trafficking.^[^ ^33^ ^]^ The binding partners, mGID1 (mammalian‐optimized Gibberellin insensitive dwarf1), and GAIs (Gibberellin insensitive), were tagged with MTs and two types of VOIs (TGN38 and LAMP3), respectively. The addition of engineered gibberellin, GA3‐AM, rapidly slowed down GAIs‐labeled post‐Golgi vesicles and lysosomes (Figure S12 and Videos S12 and S13, Supporting Information), confirming that RIVET can be triggered by various dimerization systems. Moreover, this system is not cell‐type specific, as mNeon‐FKBP‐Rab11a‐labeled recycling endosomes were inducibly halted in MDCK kidney cells (Figure S13 and Video S14, Supporting Information).

### 2.4 RIVET is Highly Specific

**Figure 2:**
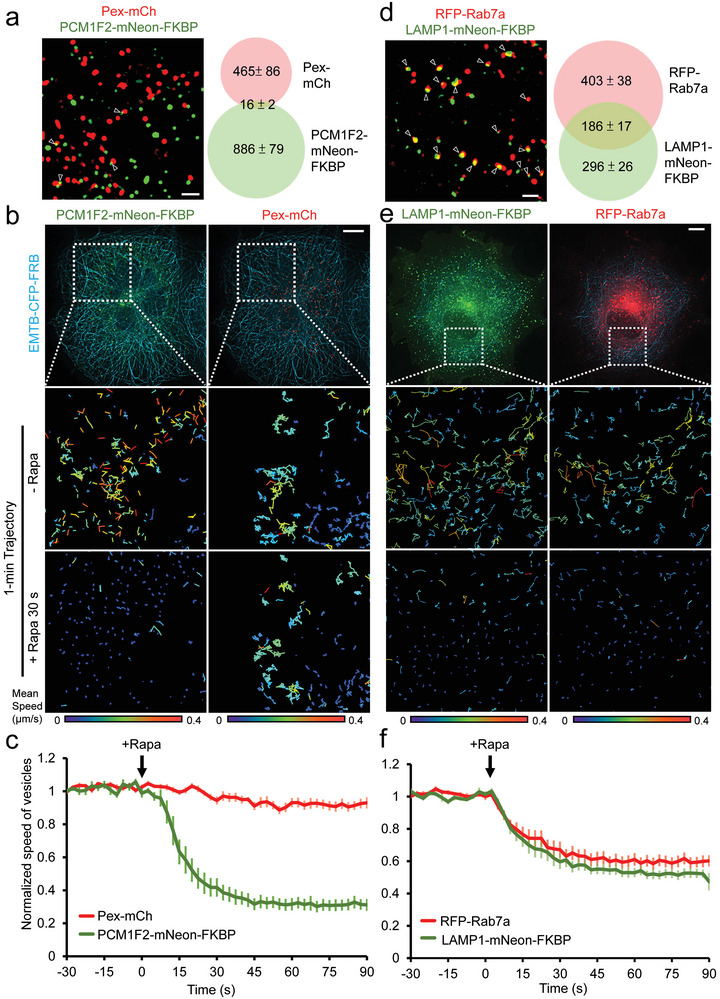
RIVET is highly specific. a,d) The fluorescence images show the distribution of the indicated vesicles in COS7 cells. Vesicles with overlapped markers are indicated by arrowheads. Scale bar, 2 µm. The Venn diagrams show the number of overlapping and non‐overlapping vesicles between the indicated vesicles. Data are shown as mean ± SEM, from 6 cells in (a) and 5 cells in (d). b) COS7 cells were transfected with EMTB‐CFP‐FRB (blue), PCM1F2‐mNeon‐FKBP (green), and Pex‐mCh (red). Trajectories show that the addition of rapamycin (Rapa, 100 nM) only halts the movement of FKBP‐positive vesicles but not Pex‐mCh‐labeled vesicles. c) The normalized mean speed of the indicated vesicles is shown in (b). *n* = 11 from 3 independent experiments. Data are shown as mean ± SEM. e) COS7 cells were transfected with EMTB‐CFP‐FRB (blue), LAMP1‐mNeon‐ FKBP (green), and RFP‐Rab7a (red). The addition of rapamycin (Rapa, 100 nM) simultaneously halts the movement of both FKBP‐positive vesicles and non‐FKBP‐labeled vesicles. f) The normalized mean speed of the indicated vesicles is shown in (e). *n* = 12 from 4 independent experiments. Data are shown as mean ± SEM.

One critical concern regarding RIVET is the potential steric hindrance of global intracellular trafficking caused by trapping VOIs on MTs. To address this, we included several non‐overlapping vesicles/organelles in this experiment. Our fluorescence imaging revealed that peroxisomes did not or only barely overlap with centriolar satellites (**Figure** **2**a). We tagged FKBP to one of these VOIs and assessed whether trapping target VOIs affected the motility of other non‐overlapping vesicles. The addition of rapamycin rapidly immobilized FKBP‐tagged centriolar satellites, causing no or minimal effect on the motility of peroxisomes (Figure 2b,c; Video S15, Supporting Information). The same strategy was used to confirm that the immobilization of centriolar satellites or post‐Golgi vesicles did not affect the dynamics of other non‐overlapping vesicles (Figure S14 and Videos S16 and S17, Supporting Information). These results strongly confirm that immobilization occurs only for the target VOIs but not for other unrelated vesicles.

Next, we investigated whether the immobilization of target vesicles perturbs their overlapping vesicles. Consistent with Rab7a's localization to late endosomes and lysosomes,^[^ ^8^ ^]^ a red fluorescent protein, RFP‐tagged Rab7a, showed significant overlap with LAMP1‐FKBP‐labeled lysosomes (Figure 2d; arrowheads). Immobilization of LAMP1‐FKBP‐labeled lysosomes using RIVET simultaneously slowed down the motility of RFP‐Rab7a‐positive vesicles (Figure 2e,f; Video S18, Supporting Information). Furthermore, Rab5b partially overlapped with Rab7a‐ and LAMP1‐vesicles, respectively (Figure S15a, arrowheads, Supporting Information). Trapping FKBP‐Rab5b endosomes also suppressed RFP‐Rab7a and LAMP1‐mCh motility (Figure S15b,c and Videos S19 and S20, Supporting Information). Such simultaneous immobilization occurred only on the FKBP‐positive vesicles, with minimal effects on vesicles without FKBP (Figure S16, Supporting Information).

Taken together, these results confirmed that RIVET‐mediated immobilization of VOIs is highly specific, only halting target VOIs and their associated vesicles with minimal impact on intracellular transport. These findings also indicate that peroxisomes, post‐Golgi vesicles, and centriolar satellites are independent transport pathways.

### 2.5 RIVET Specifically Inhibits Cargo Delivery in the Target Pathways

We next evaluated whether the immobilization of VOIs functionally suppresses specific cargo delivery. Rab5‐labeled early endosomes are required for retrograde transport.^[^ ^8^ ^]^ CTxB (Cholera toxin B subunit), a widely used probe, binds to the lipid rafts and caveolae on the cell surface and subsequently moves to the Golgi apparatus via endosomal trafficking.^[^ ^34^ ^]^ As expected, CTxB bound to the surface of control cells expressing EMTB‐CFP‐FRB and mNeon‐FKBP then moved to the perinuclear region after 30 min of incubation (arrowheads; Figure S17a,b, left, Supporting Information). Without rapamycin treatment, CTxB can move to the perinuclear region of COS7 cells expressing EMTB‐FRB and FKBP‐Rab5b via endosomal trafficking. However, rapamycin‐induced Rab5b‐vesicle immobilization attenuated the retrograde transport of CTxB (Figure S17a,b, middle, Supporting Information). Immobilization of PCM1F2‐labeled centriolar satellites did not affect CTxB transport (Figure S17a,b, right, Supporting Information), confirming the specific inhibition of cargo delivery by our system. Besides early endosomes, Rab11‐positive recycling endosomes uptake transferrin and deliver it back to the cell surface for release into extracellular space.^[^ ^8^ ^]^ Compared to control cells, immobilization of Rab11a‐ and Rab11b‐positive recycling endosomes resulted in significantly more transferrin accumulating inside the cells (Figure S17c,d, Supporting Information). These results demonstrated that immobilization of VOIs functionally suppresses the corresponding cargo delivery.

### 2.6 Rapidly and Reversibly Immobilizing VOIs by Optogenetics

**Figure 3:**
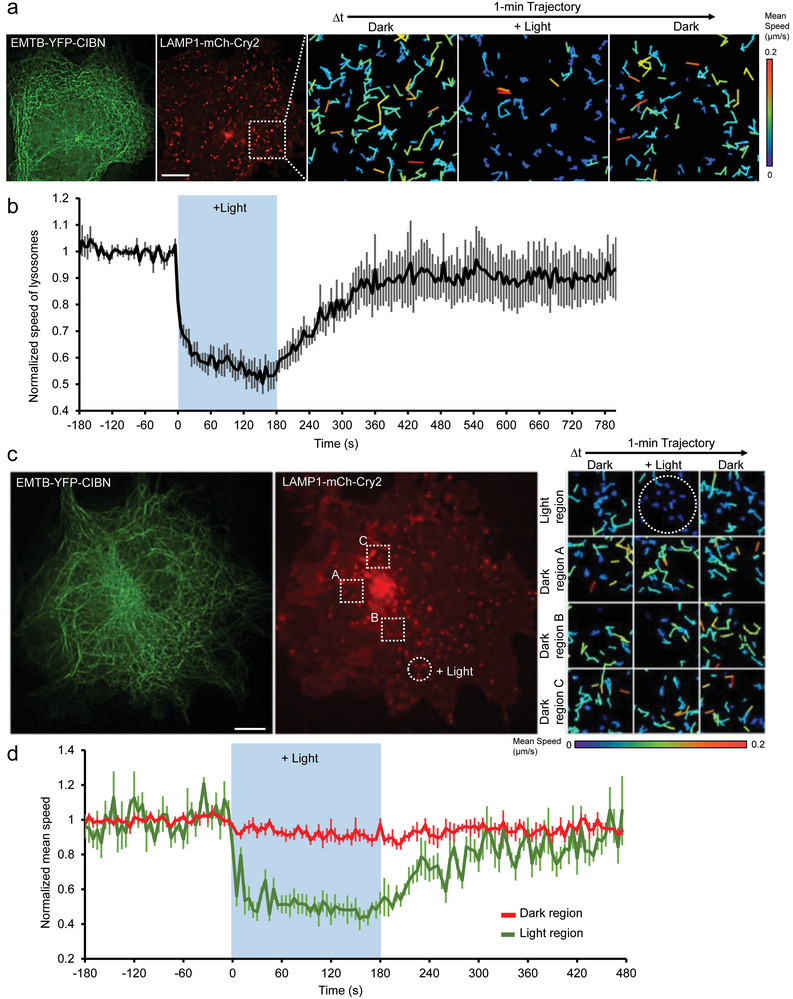
Spatiotemporally immobilizing target vesicles via the optogenetic RIVET system. a,c) COS7 cells co‐transfected with EMTB‐YFP‐CIBN (green) and LAMP1‐mCh‐Cry2 (red), were illuminated by blue light (488 nm) in the whole cell area (a), or in the indicated region locally (dotted circle) in (c). The enlarged mean speed trajectories of LAMP1‐mCh‐Cry2‐labeled lysosomes before, during, and after light stimulation in light‐illuminated and non‐stimulated regions, are shown. Scale bar, 10 µm. b,d) The normalized mean speed of LAMP1‐mCh‐Cry2‐labeled lysosomes upon global light stimulation (black curve) in cell (a), and in light‐illuminated (green curve) and non‐stimulation regions (red curve) of cell (c), is shown as mean ± SEM. *n* = 6 cells in (b) and 5 cells in (d), from 3 independent experiments.

The swift perturbation of VOIs in our system opens up the possibility of utilizing optogenetics to spatiotemporally control intracellular trafficking. Two binding partners, Cry2 (Cryptochrom2) and CIBN (N‐terminal 1–81 amino acids of CIB1), whose dimerization can be triggered by blue light illumination,^[^ ^35^ ^]^ were tagged with VOIs and MTs, respectively. The illumination of blue light on cells expressing EMTB‐YFP‐CIBN and LAMP1‐mCh‐Cry2 rapidly immobilized LAMP1‐labeled lysosomes (*t*_1/2_ on = 6.2 ± 1.2 s). The immobilized lysosomes gradually regained mobility after the termination of light illumination (*t*_1/2_ off = 68.8 ± 9.5 s; **Figure** **3**a,b; Table S1 and Video S21, Supporting Information), indicating that VOI immobilization on MTs is rapid and reversible. Furthermore, local light stimulation promptly halted the lysosome motility only in light‐illuminated regions, not in dark areas (Figure 3c,d; Video S22, Supporting Information), confirming the success of VOI perturbation at subcellular sites. The combination of precise light illumination and the rapid effect of VOI immobilization enables the spatiotemporal control of target intracellular trafficking.

### 2.7 Immobilization of Intracellular Trafficking on Selective MTs

**Figure 4:**
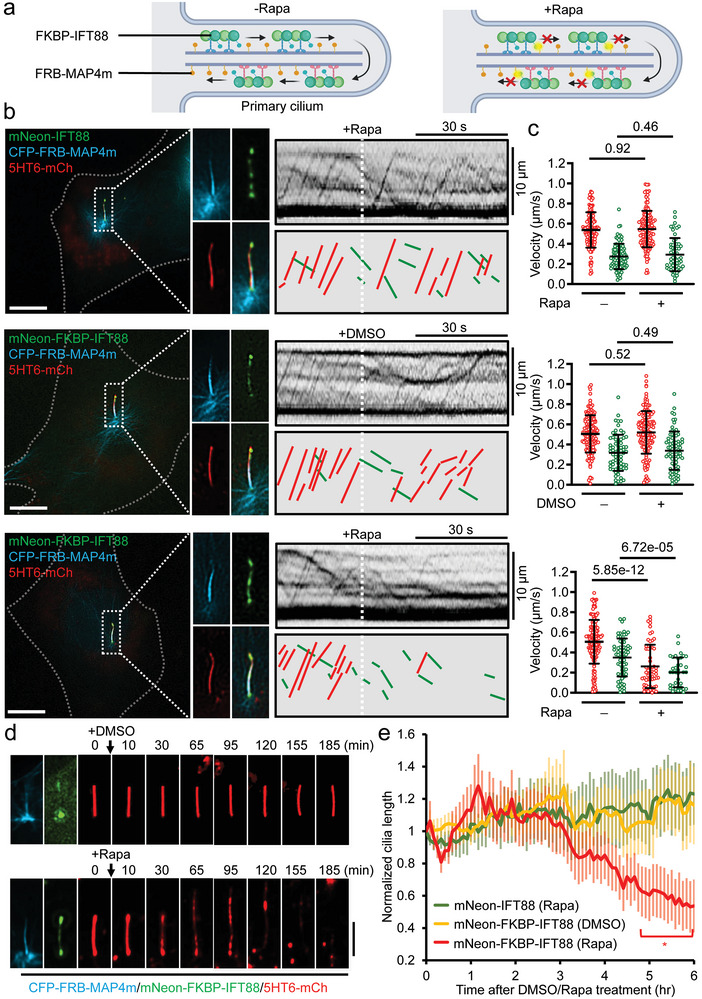
Rapid inhibition of intraflagellar transport disrupts ciliary structure. a) Schematic representation of rapidly halting intraflagellar transport (IFT) in the primary cilium. The IFT machinery and ciliary axonemes are labeled by FKBP‐IFT88 and FRB‐MAP4m, respectively. The addition of rapamycin (Rapa) immobilizes FKBP‐IFT88 onto FRB‐MAP4m‐labeled axonemes in cilia. b) NIH3T3 fibroblasts co‐transfected with 5HT6‐mCh (a ciliary membrane marker; red), CFP‐FRB‐MAP4m (an axoneme marker; blue), and mNeon‐IFT88 or mNeon‐FKBP‐IFT88‐labeled (IFT machinery markers; green) were serum‐starved for 24 h to induce ciliogenesis. Ciliated cells were then treated with 0.1% DMSO or rapamycin (Rapa, 100 nM) to trap FKBP‐tagged IFT88 onto axonemes. Kymographs depict the bidirectional movement of IFT88 puncta (red lines for anterograde movement; green lines for retrograde movement). Scale bar, 10 µm. c) The anterograde and retrograde velocities of IFT88 puncta upon 0.1% DMSO or rapamycin (Rapa, 100 nM) treatment are shown. *n* = 6, 7, and 7 cilia in upper, middle, and lower panels, respectively, from 3 to 4 independent experiments. Individual data points (velocities in anterograde and retrograde directions are shown in red and green, respectively) and the mean ± SD (black) are shown. Student's *t*‐tests were performed, with the resulting *p*‐values indicated. d) NIH3T3 fibroblasts co‐transfected with CFP‐FRB‐MAP4m (blue), mNeon‐FKBP‐IFT88 (green), and 5HT6‐mCh (red) were serum‐starved for 24 h to induce ciliogenesis. The ciliated cells were then treated with 0.1% DMSO or rapamycin (Rapa, 100 nM) and observed by live‐cell imaging for the indicated time points. Scale bar, 5 µm. e). The cilium length was measured based on the 5HT6‐mCh signal extending from the basal body. 5HT6‐mCh‐positive components that were not connected to the basal body were not included in the length measurement. The normalized length of primary cilia (red) in mNeon‐IFT88 rapamycin‐treated (green curve; *n* = 19 cilia), mNeon‐FKBP‐IFT88 DMSO treated (yellow curve; *n* = 18 cilia), and mNeon‐FKBP‐IFT88 rapamycin‐treated groups (red curve; *n* = 16 cilia), respectively, after the indicated time of DMSO/rapamycin treatment. Data are shown as mean ± SEM from 3 independent experiments. Student's *t*‐tests were performed and “*” represents *p‐*value < 0.05 between the green curve and red curve.

Cells possess several distinct MT subtypes that regulate different activities.^[^ ^29^ ^]^ Axonemal MTs act as railways for Intraflagellar transport (IFT) in primary cilia.^[^ ^36^ ^]^ Defects in IFT cause cilia to collapse, signaling malfunction, and eventually ciliopathies.^[^ ^37^ ^]^ We aim to test the possibility of IFT immobilization on axonemes using RIVET. One of the major components of IFT, IFT88,^[^ ^36^ ^]^ a ciliary membrane protein, 5HT6, and an axonemal MT‐binding protein, MAP4m,^[^ ^33^ ^]^ were used for real‐time visualizing IFT motility, cilia morphology, and axonemal MTs, respectively (**Figure** **4**a). In NIH3T3 fibroblasts, kymographs revealed that mNeon‐IFT88 processively moved in anterograde and retrograde directions along CFP‐FRB‐MAP4m‐labeled axonemal MTs. Rapamycin treatment did not affect the velocity of mNeon‐IFT88 particles in both directions (Figure 4b,c, upper panel; Video S23, left, Supporting Information). In cells transfected with mNeon‐FKBP‐IFT88 and CFP‐FRB‐MAP4m, DMSO treatment (solvent control) did not perturb IFT motility (Figure 4b,c, middle panel). However, dynamic mNeon‐FKBP‐IFT88 became static immediately after rapamycin treatment (within ≈20 s; Figure 4b,c, lower panel; Video S23, right, Supporting Information), indicating successful IFT immobilization on axonemal IFTs by RIVET. With this tool, we monitored the acute effects of IFT immobilization on cilia structure in real time. IFT immobilization reduced the width of primary cilia in the first 30 min and subsequently extended the cilia length until collapsing (Figure 4d,e; Video S24, Supporting Information). These results confirmed the successful perturbation of specific transport systems on selective cytoskeletons using RIVET and demonstrated that IFT is indeed essential to maintain the structural integrity of primary cilia.

### 2.8 Using RIVET to Perturb the Synaptic Transport In Vitro and In Vivo

**Figure 5:**
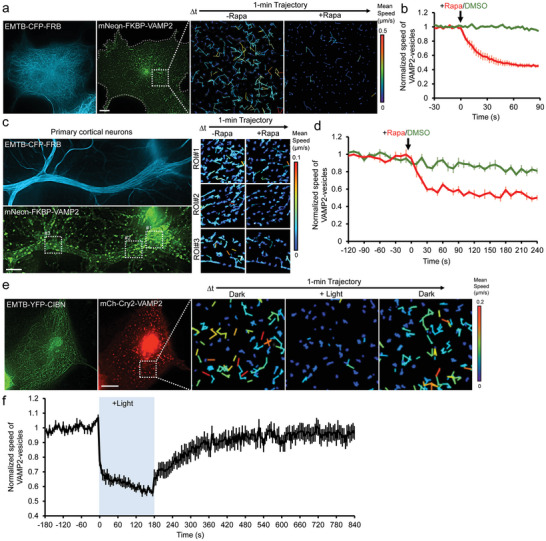
Spatiotemporal perturbation of synaptic vesicle transport in vitro. a,c) COS7 cells (a) and primary cultured cortical neurons (c) co‐expressing EMTB‐CFP‐FRB (blue) and mNeon‐FKBP‐VAMP2 were treated with rapamycin (Rapa, 100 nM) for inducible immobilization of synaptic vesicles. The enlarged mean speed trajectories of VAMP2‐positive vesicles are shown. Scale bars, 10 µm. b,d) The normalized mean speed of the synaptic vesicles upon 0.1% DMSO (green curve) or 100 nM rapamycin (red curve) treatment in COS7 cells (b) and primary culture cortical neurons (d) is shown. *n* = 11 (Rapa), and 15 cells (DMSO) in (b), 5 (Rapa), and 6 neurons (DMSO) in (d), from 3 independent experiments. Data are shown as mean ± SEM. e) COS7 cells co‐transfected with EMTB‐YFP‐CIBN (green) and mCh‐Cry2‐VAMP2 (red) were temporally illuminated by blue light in whole cells. The enlarged mean speed trajectories of VAMP2‐positive vesicles are shown. Scale bar, 10 µm. f) The normalized mean speed of VAMP2‐positive vesicles upon temporal blue light illumination (blue) in (e). Data are shown as mean ± SEM. *n* = 6 cells from 3 independent experiments.

Synaptic vesicles deliver neurotransmitters along MTs from the cell body (soma) to synaptic sites in neuronal cells. Defects in such transport cause a wide range of neurodegenerative diseases^[^ ^38^ ^]^ A tool enabling the spatiotemporal manipulation of synaptic vesicle transport is highly desirable to uncover the etiology of relative diseases. VAMP2 (Vesicle‐associated membrane protein 2), a target protein of synaptic vesicles, was tagged with FKBP. To prove the concept, mNeon‐FKBP‐VAMP2‐labeled vesicles in COS7 cells can be rapidly immobilized on EMTB‐labeled MTs by RIVET (*t*_1/2_: 19.2 ± 4.4 s; **Figure** **5**a,b; Video S4, Supporting Information). The same mechanism can be also successfully achieved in primary culture cortical neurons (*t*_1/2_: 13.6 ± 3.4 s; Figure 5c,d; Video S25, Supporting Information). To perturb synaptic vesicle motility with better spatiotemporal resolution, we optogenetically trapped Cry2‐VAMP2‐labeled vesicles on EMTB‐CIBN‐bound MTs in a reversible manner (*t*_1/2_ on: 7.0 ± 1.9 s; *t*_1/2_ off: 68.1 ± 15.2 s; Figure 5e,f; Video S26, Supporting Information).

**Figure 6:**
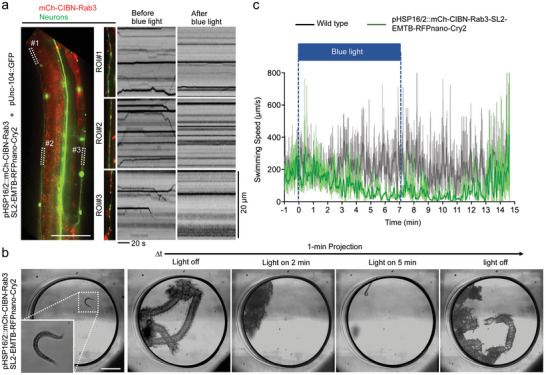
Using light to manipulate synaptic vesicle transport in vivo. a) The fluorescence image of transgenic *C. elegans* carrying pHSP16/2::mCh‐CIBN‐Rab3‐SL2‐EMTB‐RFPnano‐Cry2 and pUNC104::GFP is shown. Neuron cells and synaptic vesicles were labeled by GFP (green) and mCh‐CIBN‐Rab3 (red), respectively. Three regions of interest (ROI) were highlighted by dotted squares. Scale bar, 50 µm. Kymographs were used to show the dynamics of mCh‐CIBN‐Rab3 before and after 3 min of blue light stimulation. Scale bars of kymographs, 20 µm. b) The 1 min projection of time‐lapse images of transgenic *C. elegans* expressing mCh‐CIBN‐Rab3‐SL2‐EMTB‐RFPnano‐Cry2 before, during, and after blue light illumination in a thrashing assay. Scale bar, 1 mm. c) The quantified swimming speed of wild‐type (black curve) and pHSP16/2::mCh‐CIBN‐Rab3‐SL2‐EMTB‐RFPnano‐Cry2 (green curve) transgenic worms is shown. The time period of blue light illumination is indicated by a dark blue rectangle. The curves are shown as rolling averages. *n* = 10 worms in both wild‐type and pHSP16/2::mCh‐CIBN‐Rab3‐SL2‐EMTB‐RFPnano‐Cry2 transgenic worms, from 4 independent experiments.

Previous studies have perturbed synaptic transmission using different methods, which attenuate animal locomotion.^[^ ^22^, ^23^ ^]^ To assess the effects of synaptic vesicle immobilization on animal behavior, we generated transgenic *C. elegans* co‐expressing EMTB‐Cry2 and CIBN‐Rab3 in which Rab3 targets synaptic vesicles (**Figure** **6**).^[^ ^39^ ^]^ As expected, light illumination halted the Rab3‐labeled synaptic vesicles in transgenic worms (Figure 6a; Video S27, Supporting Information). It did not affect the swimming cycle of transgenic *C. elegans* in the early phase, and then gradually slowed the worms down after ≈5 min of light stimulation. The paralyzed worms gradually restored their movement to the basal level when the light was ceased. Such light stimulation did not affect the swimming of wild‐type *C. elegans* (Figure 6b,c; Video S28, Supporting Information). These results indicate RIVET can modulate animal behavior by perturbation of synaptic transmission in a reversible manner.

### 2.9 Inhibition of Receptor‐Mediated Endocytosis to Attenuate SARS‐CoV2 Entry

**Figure 7:**
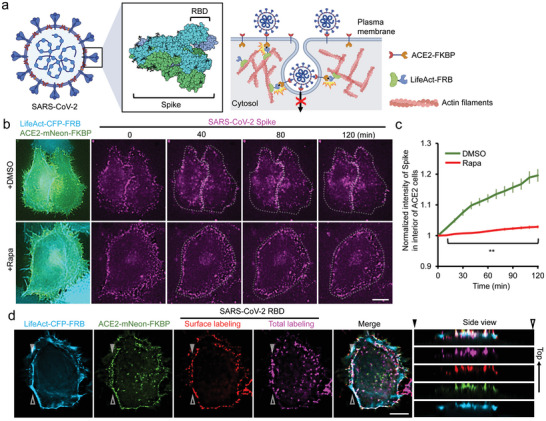
Precise perturbation of endocytosis to inhibit virus entry. a) Schematic representation of the SARS‐CoV2 structure and the experimental design to trap ACE2 on LifeAct‐FRB‐labeled actin filaments to prevent virus entry. The enlarged protein structures of the Spike protein and RBD (receptor binding domain) are shown. The graph was created using BioRender. b) HeLa cells stably expressing ACE2 were transfected with the indicated constructs. After 5 min of 0.1% DMSO or 100 nM rapamycin (Rapa) treatment, transfected cells were incubated with SARS‐CoV‐2 spike‐Alexa Fluor 647 (purple; 1.2 ng µl^−1^) for 5 min. Excess spike‐Alexa Fluor 647 was washed out, and its internalization for the indicated times is shown. Scale bar, 10 µm. c) The normalized intensity of spike‐Alexa fluor 647 in the interior of cells (the region 3 µm inward from the cell periphery) transfected and treated with the indicated conditions is shown. *n* = 49 (DMSO), and 50 cells (Rapa) from 3 independent experiments. Data are shown as mean ± SEM. Significance was determined by the Student's *t*‐test. “**” represents *p* < 0.01. d) Fluorescence images of SARS‐CoV‐2 RBD protein were labeled by different colors of antibodies to distinguish surface (red) and total (purple) RBD proteins in ACE2‐positive HeLa cells transfected with LifeAct‐CFP‐FRB (blue) and ACE2‐mNeon‐FKBP (green) after 1 h of RBD treatment. A side view of the indicated regions between arrowheads is shown on the right. Scale bar, 10 µm.

SARS‐CoV2 (Severe acute respiratory syndrome coronavirus 2), the causative pathogen of COVID‐19, is known to bind to the ACE2 receptor (Angiotensin‐converting enzyme 2) via RBD (receptor binding domain) of virus’ Spike protein, which triggers ACE2‐mediated endocytosis for subsequent infection processes (**Figure** **7**a).^[^ ^39^ ^]^ In this experiment, the fluorescently labeled Spike protein derived from SARS‐CoV2 was incubated with HeLa cells stably expressing human ACE2. Live‐cell imaging showed that Spike efficiently bound to the cell surface and then gradually moved to the interior of cells in ACE2‐positive HeLa cells (DMSO treated groups in Figure 7b,c, green curve; Video S29, upper panel, Supporting Information). Since actin filaments at the cell cortex are required to initiate the invagination of endocytic components,^[^ ^40^ ^]^ we attempted to stall the cytosolic domain of ACE2 on actin filaments. To test whether dimerization can be triggered in ACE2‐positive components, mNeon‐FKBP was tagged to the cytosolic domain of ACE2, and the resulting fusion protein was co‐expressed with cytosolic CFP‐FRB protein. The addition of rapamycin rapidly translocated cytosolic CFP‐FRB onto ACE2‐mNeon‐FKBP labeled plasma membrane (Figure S18 and Video S30, Supporting Information). FRET imaging showed that dimerization between ACE2 and actin filaments can be triggered in cell peripheries (Figure S19 and Video S31, Supporting Information). Moreover, trapping ACE2 at cortical actin blocked ≈89% of SARS‐CoV2 spike internalization after 2 h of incubation (Figure 7b,c; Video S29, lower panel, Supporting Information). Surface labeling assay also confirmed that most of the SARS‐CoV2 RBD retained on the surface of cells co‐expressing LifeAct‐FRB and ACE2‐FKBP upon rapamycin treatment (Figure 7d). These results indicate that using RIVET to trap ACE2 on cortical actin blocks ACE2‐mediated endocytosis as well attenuates SARS‐CoV2 spike internalization.

## 3 Discussion

RIVET is a highly specific system, as trapping target vesicles on MTs does not affect the motility of other unrelated or non‐overlapping vesicles (Figure 2a–c, left; Figure S14 and Videos S15–S17, Supporting Information). A previous study also found that light‐induced large vesicle clusters do not affect the motility of intracellular transport,^[^ ^21^ ^]^ implying that distinct vesicles move independently. There are two possible scenarios to explain why vesicles trapped on MTs do not act as “roadblocks” for other MT‐dependent trafficking: 1) Unrelated vesicles share the same population of MTs as their railways. MTs form bundles to provide a large surface area of railways that is not fully occupied by trapped vesicles or clusters. 2) Non‐overlapping vesicles always run on distinct populations of MT subtypes. Trapping target vesicles on their own MT subtypes does not affect others’ railways. Railway‐specific vesicular trafficking has been reported previously. For example, anterograde and retrograde IFT trains move on distinct sites of ciliary axonemes.^[^ ^41^ ^]^ Although the evidence is indirect, MTs undergo different post‐translational modifications and have preferential affinities to different types of motor proteins that may act as railways for distinct transport pathways.^[^ ^42^ ^]^ Whether non‐overlapping vesicles run along distinct MT subtypes needs to be further evaluated. Recently, many biosensors that bind to specific MT subtypes have been developed.^[^ ^29^, ^43^ ^]^ With these biosensors, the efficiency of trapping target vesicles on specific MT subtypes can be quantitatively assessed using RIVET.

The essential roles of IFT in the structural integrity of primary cilia have been well studied. Genetic depletion of IFT88 fully inhibits the ciliogenesis in cultured cells and animals.^[^ ^36^, ^44^, ^45^ ^]^ To understand the causative roles of IFT in cilia, Engelke et al. established an elegant approach to halt IFT using an engineered inhibitable kinesin‐2 motor.^[^ ^46^ ^]^ Such a strategy approximately takes 5 min to slow down the IFT machinery in cilia, resulting in progressive cilia shortening. Our results showed that RIVET can completely halt IFT within 20 s (Figure 4b, lower panel; Video S23, right, Supporting Information). Taking advantage of this acute inhibition of IFT, we real‐time monitored the direct effects of IFT inactivation on the ciliary structure. The cilia rapidly become thinner and then elongate in length until they collapse (Figure 4d,e; Video S24, Supporting Information). Such thin and long cilia have been also observed in Cenpj‐depleted neural progenitor cells, which causes microcephaly in mice.^[^ ^47^ ^]^ Since RIVET is a genetic‐based tool, such acute IFT inhibition in target tissues is promising to decipher the causative roles of IFT and cilia in the etiology of related disorders.

To spatiotemporal perturb synaptic vesicle transmission, several studies used optogenetic tools to cluster synaptic vesicles, which results in inhibition of synaptic vesicle release.^[^ ^22^, ^23^ ^]^ Clustering of synaptic vesicles inhibits locomotion in *C. elegans* and zebrafish within seconds.^[^ ^23^ ^]^ Although clustering and immobilization of synaptic vesicles can be triggered under comparable kinetics (Figures 5 and 6a; Table S1 and Videos S4, S25–S27, Supporting Information),^[^ ^22^, ^23^ ^]^ RIVET‐mediated synaptic vesicle trapping on MTs takes significantly longer time (≈5 min) to attenuate animal locomotion compared to synaptic vesicle clustering systems (Figure 6b,c; Video S28, Supporting Information). The readily releasable pool of synaptic vesicles in synapses needs to be replenished by synaptic vesicle transport along MTs, serving as a rate‐limiting mechanism for high‐frequency neurotransmission.^[^ ^38^ ^]^ In contrast to such immediate inhibition of synaptic vesicle release by optogenetic clustering, trapping synaptic vesicles on MTs halts the replenishment of the reserve pool of neurotransmitters. This results in a delayed functional inhibition after the readily releasable synaptic vesicles are depleted. The paralyzed *C. elegans* restore locomotion gradually in the dark (Figure 6b,c; Video S28, Supporting Information), implying that inhibition of synaptic vesicle transmission is reversible. A previous study also found that the disturbance of actin filaments and MTs affects the fast‐recycling and slow‐recycling of synaptic vesicles, respectively.^[^ ^48^ ^]^ Whether immobilization of synaptic vesicles in actin filaments or MTs perturb animal locomotion in different kinetics is worthy of studying using RIVET.

## 4 Experimental Section

### Cell Culture

COS7, HeLa, NIH3T3, MDCK cells, and ACE2‐stable HeLa cells^[^ ^49^, ^50^ ^]^ were maintained at 37 °C, 5% CO_2_, and 95% humidity in Dulbecco's modified Eagle's medium (DMEM) supplemented with 10% fetal bovine serum (FBS), penicillin, and streptomycin (Corning). For the primary culture of cortical neurons, the cortex from Day 18 to 19 rat embryonic brains was dissected into small pieces (1–2 mm^3^). These tissues were washed with Hank's balanced salt solution without calcium and magnesium (HBSS/wo Ca^2+^–Mg^2+^) to remove contaminants and vessel debris. The tissues were then digested in a sterile‐filtered papain solution (0.6 mg mL^−1^ papain, 0.2 mg mL^−1^ l‐cysteine, 0.6 mg mL^−1^ DNase I, 1.5 mM CaCl_2_, and 0.5 mM EDTA in HBSS) at 37 °C in a water bath for 15–30 min with gentle shaking every 5 min. The enzymatic reaction was stopped by treating with a sterile‐filtered inactivation solution (composed of 10 mL serum media, 25 mg bovine albumin, and 25 mg trypsin inhibitor; 10 mL serum media includes 0.5 mL FBS, 38.3 mg glucose, and 9.5 mL MEM with Earle's salts without L‐glutamine) at 37 °C in a water bath for 2 min. After aspirating the inactivation solution, the tissues were gently triturated 20–30 times into a suspension of neurons using a 10 mL plastic pipette. A 40 µm cell strainer was used to remove undigested tissues, and the suspended cells were collected by centrifugation at room temperature at 1000xg for 5 min. The cells were resuspended in a Neurobasal (NB) medium containing 1 × GlutaMAX supplement, 12.5 µM glutamate, 1 × penicillin‐streptomycin, and B27 supplement. A cell density of 4 × 10^5^ cells/well was seeded onto poly‐l‐lysine‐coated 6‐well plates for further incubation. Every four days, one‐third of the fresh NB/B‐27 medium without glutamate was added to maintain the cell health.

### DNA Constructs

Several vesicular markers were cloned into mNeon‐FKBP, mNeon‐GAIs, or mCh‐Cry2 vectors^[^ ^30^, ^31^ ^]^ to generate the following constructs: LAMP1‐mNeon‐FKBP, PCM1F2‐mNeon‐FKBP, Pex‐mNeon‐FKBP, mNeon‐FKBP‐Rab5b, mNeon‐FKBP‐Rab11a, mNeon‐FKBP‐Rab11b, mNeon‐FKBP‐VAMP2, TGN38‐mNeon‐FKBP, mNeon‐FKBP‐Rab37, mNeon‐FKBP‐IFT88, ACE2‐mNeon‐FKBP, TGN38‐mNeon‐GAIs, LAMP3‐mNeon‐GAIs, LAMP1‐mCh‐Cry2, and mCh‐Cry2‐VAMP2. The gene “mCh‐CIBN‐Rab3‐SL2‐EMTB‐RFPnano‐Cry2” was synthesized and subsequently subcloned into a vector under the phSP16/2 promoter. The MAPTau gene was obtained from mNeon‐MAPTau (a gift from Dorus Gadella; Addgene plasmid #137 808) and subcloned into the CFP‐FRB construct. Each construct was transformed into competent cells, and single clones were isolated for DNA purification. All DNA constructs were verified through DNA sequencing. The design of the constructs (Figure S20, Supporting Information) and their protein sequences used in this study are provided in the Supporting Information.

### DNA Transfection and AAV Infection

HeLa, NIH3T3, and MDCK cells were transfected using FuGENE HD (Promega), while COS7 cells were transfected with Turbofect transfection reagent (Thermo Fisher Scientific). After transfection, the cells were incubated for 24–28 h before imaging and other experiments were conducted. The AAV9/CB‐EMTB‐CFP‐FRB and AAV9/CB‐mNeon‐FKBP‐VAMP2 constructs were produced at the AAV core facility in Academia Sinica, Taiwan, with a titer exceeding 5.0E + 13 vg mL^−1^. To initiate the experiment, transfer part of the culture media into a 15 mL plastic tube, leaving ≈1–1.5 mL of media in the well. Add the viral stock to the remaining media in the well and incubate the samples at 37 °C in a 5% CO_2_ incubator for 24 h. After the incubation period, replace the virus‐containing media with the previously set aside culture media from the 15 mL plastic tube. Monitor the fluorescence signal of gene overexpression for 7 days post‐infection.

### Live‐Cell Imaging

Transfected cells were cultured on poly(D‐lysine)‐coated glass coverslips (Hecht Assistent) or in borosilicate glass Lab‐Tek eight‐well chambers (Nunc). Cells were treated with either 100 nM rapamycin or 100 µM GA3‐AM to rapidly induce protein dimerization, or with 0.1% DMSO as a solvent control during imaging. Live‐cell imaging was performed using a Nikon T1 inverted fluorescence microscope equipped with a 60× oil objective and a Prime camera (Photometrics), all maintained at 37 °C and 5% CO_2_ using a heated stage (Live Cell Instrument). Rapid recruitment of VOIs was imaged at intervals of either 2.5 or 5 s. For IFT imaging, NIH3T3 cells transfected with the indicated constructs were serum‐starved for 24 h to induce ciliogenesis. The ciliated cells expressing IFT88‐mNeon were imaged every 200 ms for the specified duration^[^ ^33^ ^]^ Images were captured using Nikon NIS‐Elements AR software and processed with the Nikon deconvolution module. FRET imaging utilized CFP, YFP, and CY‐FRET filters, with the FRET/CFP ratio calculated by the FRET module in the NIS‐Elements AR software.^[^ ^51^ ^]^ Kymographs and time‐lapse videos were also prepared using Nikon NIS‐Elements AR software.

### Tracking Endomembrane Vesicles

Transfected COS7 cells, HeLa cells, and MDCK cells were imaged at intervals of 2.5 or 5 s. Vesicle trajectories were analyzed using Fiji software with the Trackmate plugin. The displacement and velocity of VOIs were tracked using the DoG detector and Simple LAP tracker. Trajectory data were processed using Moment Scaling Spectrum analysis (software available at https://www.utsouthwestern.edu/labs/jaqaman/software/), with parameters set to: blob diameter (0.8–1 µm), linking max distance (1.2 µm), gap‐closing distance (2 µm), and gap‐closing max frame gap (1).

### FRAP Assay

Fluorescence Recovery After Photobleaching (FRAP) was performed using a confocal laser scanning microscope equipped with a 60x oil objective (Zeiss LSM780). The experiment utilized the “bleaching,” “time series,” and “regions” functions of ZEN software. Prior to bleaching, an initial image was captured to establish a baseline for fluorescence intensity. The region of interest (ROI) was then photobleached with 100% 488 nm laser power and allowed to recover for the indicated time. Images of the ROI were subsequently captured every 30 s for a total of 170 s following the bleaching.

### CTxB and SASR‐CoV2 Spike Endocytosis Assay

Transfected cells were treated with 0.1% DMSO or 100 nM rapamycin for 5 min before incubation with CTxB‐Alexa Fluor 555 (1 µg mL^−1^; Thermo Fisher Scientific, C34776) or recombinant SARS‐CoV‐2 Spike protein‐Alexa Fluor 647 (1.2 ng µl^−1^; R&D Systems; AFR10878) at 37 °C for 5 min. After incubation, cells treated with CTxB were washed to remove unbound CTxB and fixed with 4% paraformaldehyde (Electron Microscopy Sciences) at room temperature for 15 min, followed by imaging. To analyze the radial distribution of CTxB‐Alexa Fluor 555, the Broad Institute's Cell Profiler 3.0 software was used to generate a workflow.^[^ ^52^, ^53^ ^]^ This workflow measured the mean fractional intensity of the CTxB signal within the cell using the program's radial distribution module, which divided the cell into 20 equal‐sized radial bins extending from the center of the nucleus to the cell periphery. In the SARS‐CoV‐2 internalization experiment, cells bound with the Spike protein were monitored using live‐cell imaging over the specified duration. The intensity of Spike protein in the region 3 µm inward from the cell periphery was measured by Nikon NIS element software.

### Labeling SARS‐CoV2 RBD Protein on the Cell Surface and Within Entire Cells

ACE2 positive HeLa cells were incubated with 1.2 ng µl^−1^ SARS‐CoV‐2 spike RBD protein (Spike subunit 1, Fc tagged recombinant protein; Sino Biological Inc.; 40591‐V02H) in serum‐free medium at 37 °C for 5 min. After this incubation, the unbound RBD protein was washed out using a medium containing 4% FBS. The cells were then incubated in a 4% FBS medium at 37 °C for 1 h. Following this, cells were fixed with 4% paraformaldehyde (Electron Microscopy Sciences) at room temperature for 15 min and subsequently incubated in a blocking solution (phosphate‐buffered saline with 2% bovine serum albumin) for 30 min at room temperature. To label the SARS‐CoV‐2 RBD protein on the cell surface, cells were incubated with Alexa Fluor 594 goat anti‐human IgG (H+L) antibody (1:1000; Invitrogen) for 1 h at room temperature. The cells were then permeabilized with 0.1% Triton X‐100 and incubated again in a blocking solution for 30 min at room temperature. Afterward, cells were incubated with Alexa Fluor 647 goat anti‐human IgG (H+L) antibody (1:1000; Invitrogen) for 1 h at room temperature to label the SARS‐CoV‐2 RBD protein within the entire cells.

### Transferrin Recycling Assay

Transfected COS‐7 cells were serum‐starved for 3 h to enhance the uptake of transferrin probes in the following steps. To uptake fluorescent transferrin, cells were incubated with Alexa Fluor 594‐conjugated Transferrin (Transferrin‐594; Invitrogen; T13343) diluted in serum‐containing medium (1:200) at 37 °C for 5 min. After washing away unbound Transferrin‐594, cells were then incubated in serum‐containing medium at 37 °C for 30 min and fixed by incubation with paraformaldehyde (Electron Microscopy Sciences) diluted in PBS at room temperature for 15 min.

### Establishment of Transgenic Worms

*C. elegans* strains were cultivated on nematode growth medium (NGM) and fed with E. coli strain OP50‐1, following standard methods. The animals were grown at room temperature and kept in the dark. Transgenic animals were created by microinjecting plasmid DNA containing pHSP16/2::mCh‐CIBN‐Rab3‐SL2‐EMTB‐RFPnano‐Cry2 and pUNC‐104::GFP (a neuronal marker) into the gonads of wild‐type Bristol N2 animals using standard procedures.

### Photostimulation in Living Cell Experiment

COS7 cells were plated on poly(D‐lysine)‐coated coverslips and cultured in six‐well plates (Thermo Scientific) for 24 h. Local photostimulation was conducted using a Nikon T1 inverted fluorescence microscope equipped with a digital micromirror device (Polygon 400; MIGHTEX) and a 488 nm light source. Cells were illuminated with blue light (488 nm; 1 s on/1 s off; 2.4 mW mm^−^^2^) for the indicated duration. During photostimulation, images were captured using Nikon Element AR software.

### Behavioral Assays

After 1 h of heat shock at a specified temperature (33 °C) in a completely dark environment, young adult worms were carefully transferred onto plain agar‐rich slides using 3 µl of M9 buffer, and covered with a coverslip, all while avoiding light exposure. The worms were then allowed to acclimate to swimming behavior in darkness for 15 min. Subsequently, the slides were positioned under a Nikon T1 inverted fluorescence microscope equipped with a digital micromirror device (Polygon 400; MIGHTEX) and a 488 nm light source. The worms were exposed to blue light (488 nm) for 7 min (6.9 mW mm^−^^2^). Videos of the swimming behavior were automatically recorded for 15 min, including 1 min before, 7 min during, and 8 min after light stimulation. The swimming speed of the light‐stimulated worms was analyzed using the commercial software WormLab (MBF Bioscience).

### Statistical Analysis

Whether variances were equal or not with the F‐test were first determined and then used the unpaired two‐tailed Student's *t*‐test to calculate *P*‐values via PRISM 10 software. A *P*‐value of < 0.05 indicated a significant difference, and *p* < 0.01 indicated a highly significant difference.

## Conflict of Interest

The authors declare no conflict of interest.

## Author Contributions

S.C.C., N.J.Z., and G.Y.L. contributed equally to this work. S.C.C. and Y.C.L. designed the experiments. S.C.C., G.Y.L., C.L.K., H.C., B.H.W., P.C.S., and Y.C.L. conducted the vesicle experiments in cell lines. Y.C.C. performed the FRET imaging. H.C.W. conducted the experiments on primary cultured neurons under the supervision of P.H.C. and Y.C.L. S.C.C. and Y.F. conducted the Spike and RBD experiments with assistance from Y.T.C. and L.H.C.W. C.Y.C. conducted the IFT experiment. Y.L.T., C.H.S., and W.H.C. analyzed the data in cilia experiments. N.J.Z. and T.Y.L. performed the worm experiments with assistance from O.B. and O.I.W. J.B.Y. analyzed worm behavior under the supervision of C.H.C. Y.C.W. helped in exocytic vesicle experiments. T.Y.L. performed the FRAP experiment. S.C.C. and Y.C.L. wrote the paper with input from all authors.

## Supporting information



Supporting Information

Supplemental Video 1

Supplemental Video 2

Supplemental Video 3

Supplemental Video 4

Supplemental Video 5

Supplemental Video 6

Supplemental Video 7

Supplemental Video 8

Supplemental Video 9

Supplemental Video 10

Supplemental Video 11

Supplemental Video 12

Supplemental Video 13

Supplemental Video 14

Supplemental Video 15

Supplemental Video 16

Supplemental Video 17

Supplemental Video 18

Supplemental Video 19

Supplemental Video 20

Supplemental Video 21

Supplemental Video 22

Supplemental Video 23

Supplemental Video 24

Supplemental Video 25

Supplemental Video 26

Supplemental Video 27

Supplemental Video 28

Supplemental Video 29

Supplemental Video 30

Supplemental Video 31

## Data Availability

The data that support the findings of this study are available from the corresponding author upon reasonable request.
