# Supplementary material for: Precise Control of Intracellular Trafficking and Receptor‐Mediated Endocytosis in Living Cells and Behaving Animals
Source: Adv Sci (Weinh). 2024 Oct 14;11(45):2405568. doi: 10.1002/advs.202405568 (PMC11615828; doi:10.1002/advs.202405568)
Supplement: Supplementary file 1 — Supporting Information [file ADVS-11-2405568-s015.pdf]

ADVANCED SCIENCE Open Access

Supporting Information

for *Adv. Sci.*, DOI 10.1002/advs.202405568

Precise Control of Intracellular Trafficking and Receptor-Mediated Endocytosis in Living Cells and Behaving Animals

*Shiau-Chi Chen, Neng-Jie Zeng, Grace Y. Liu, Hsien-Chu Wang, Tzu-Ying Lin, Yi-Ling Tai, Chiao-Yun Chen, Yin Fang, Yi-Chien Chuang, Ching-Lin Kao, Hsuan Cheng, Bing-Huang Wu, Pin-Chiao Sun, Odvogmed Bayansan, Yu-Ting Chiu, Chi-Hsuan Shih, Wen-Hong Chung, Jia-Bin Yang, Lily Hui-Ching Wang, Po-Han Chiang, Chun-Hao Chen, Oliver I. Wagner, Yi-Ching Wang and Yu-Chun Lin**

Supporting Information

Precise Control of Intracellular Trafficking and Receptor-Mediated Endocytosis in Living Cells and Behaving Animals

Shiau-Chi Chen[1*], Neng-Jie Zeng[1*], Grace Y. Liu[1*], Hsien-Chu Wang[1], Tzu-Ying Lin[1], Yi-Ling Tai[1], Chiao-Yun Chen[1], Yin-Fang[1], Yi-Chien Chuang[1], Ching-Lin Kao[1], Hsuan Cheng[1], Bing-Huang Wu[1], Pin-Chiao Sun[1], Odvogmed Bayansan[2], Yu-Ting Chiu[2], Chi-Hsuan Shih[1], Wen-Hong Chung[1], Jia-Bin Yang[3], Lily Hui-Ching Wang[2], Po-Han Chiang[4], Chun-Hao Chen[3], Oliver I. Wagner[2,5], Yi-Ching Wang[6], Yu-Chun Lin[1,5,7#]

Supplementary Figures and Videos:
Content:

Table S1
Figure S1
Figure S2
Figure S3
Figure S4
Figure S5
Figure S6
Figure S7
Figure S8
Figure S9
Figure S10
Figure S11
Figure S12
Figure S13
Figure S14
Figure S15
Figure S16
Figure S17
Figure S18
Figure S19
Figure S20

Video S1
Video S2
Video S3
Video S4
Video S5
Video S6
Video S7
Video S8
Video S9
Video S10
Video S11
Video S12
Video S13
Video S14
Video S15
Video S16
Video S17
Video S18
Video S19
Video S20

Video S21
Video S22
Video S23
Video S24
Video S25
Video S26
Video S27
Video S28
Video S29
Video S30
Video S31

Supporting information of protein sequence

Table S1. The kinetic and inhibition efficiency of different VOIs in RIVET. The data was obtained using EMTB-FRB or EMTB-CIBN, except for two conditions that utilized FRB-MAPTau* and FRB-MAP4m#. Data are presented as mean ± SEM.

Vesicle of Interests	Targeting markers	$t_{1/2}$ (CID)	Velocity reduction (CID)	$t_{1/2}$ of on and off rate (LID)	Velocity reduction (LID)
Lysosomes	LAMP1	11.0 ± 3.1 s	45.0 ± 4.0 %	On: 6.2 ± 1.2 s<br>Off: 68.8 ± 9.5 s	41.2 ± 4.8 %
	LAMP3	8.3 ± 1.5 s	54.2 ± 1.7 %	N/A	N/A
Peroxisomes	Pex	20.3 ± 5.0 s	56.1 ± 4.4 %	N/A	N/A
Endosomes	Rab5b	11.8 ± 4.0 s	52.5 ± 4.4 %	N/A	N/A
Post-Golgi vesicles	TGN38	9.0 ± 1.3 s<br>17.0 ± 2.2 s*	47.4 ± 4.0 %<br>43.6 ± 1.6 %*	N/A	N/A
Recycling endosomes	Rab11b	12.5 ± 2.8 s	44.4 ± 3.2 %	N/A	N/A
Synaptic vesicles	VAMP2	19.2 ± 4.4 s	54.9 ± 2.5 %	On: 7.0 ± 1.9 s<br>Off: 68.1 ± 15.2 s	47.2 ± 1.5 %
Centriolar satellites	PCM1F2	10.5 ± 2.5 s	63.5 ± 5.0 %	N/A	N/A
Exocytosis vesicles	Rab37	12.3 ± 3.6 s	52.9 ± 2.5 %	N/A	N/A
IFT components	IFT88	N/A	Anterograde:<br>58.0 ± 2.8 %#<br>Retrograde:<br>42.3 ± 2.4 %#	N/A	N/A

Figure S1

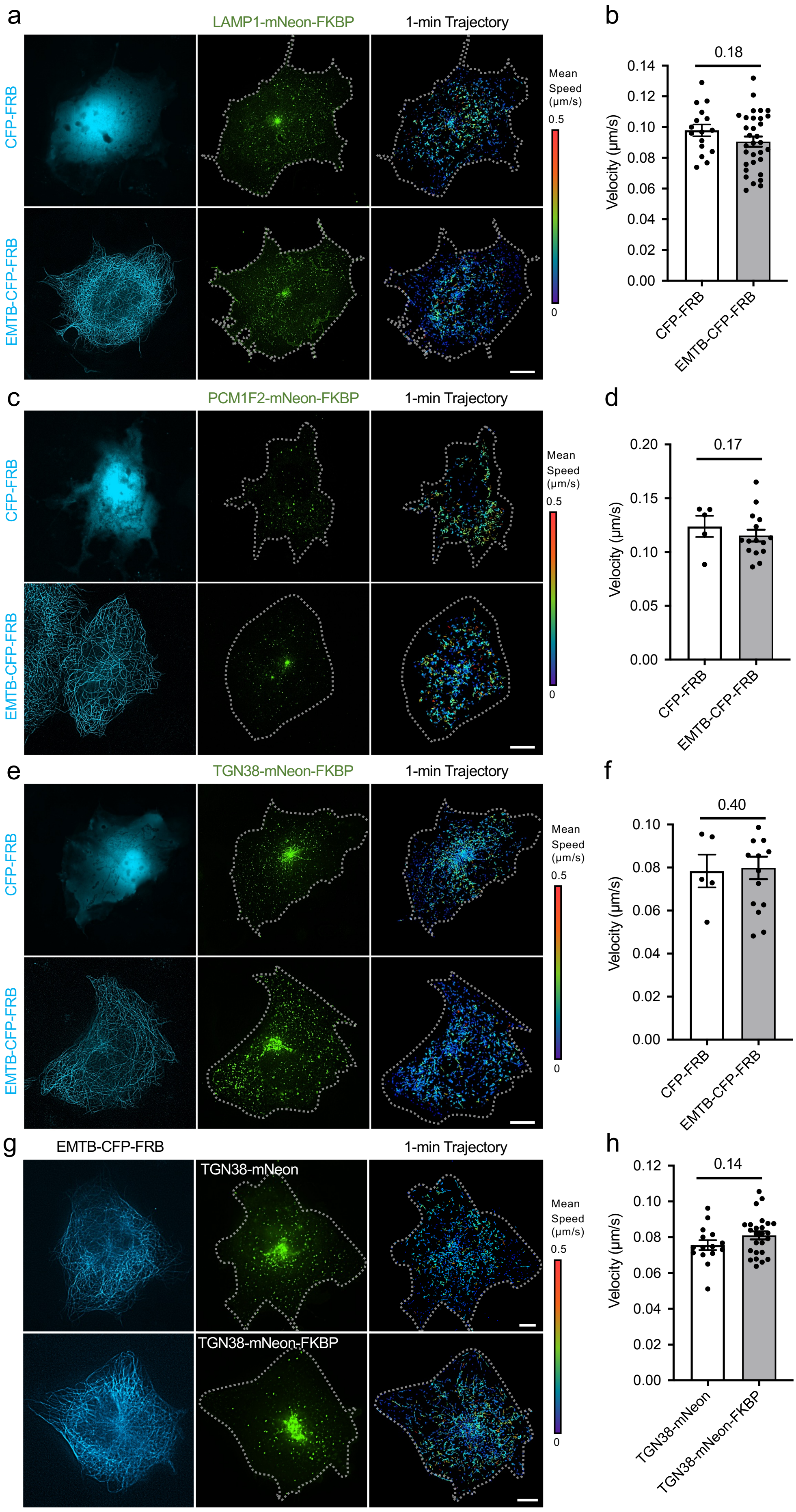

a
LAMP1-mNeon-FKBP
1-min Trajectory
CFP-FRB
EMTB-CFP-FRB
Mean Speed (μm/s)
0.5
0
b
0.18
Velocity (μm/s)
0.14
0.12
0.10
0.08
0.06
0.04
0.02
0.00
CFP-FRB
EMTB-CFP-FRB
c
PCM1F2-mNeon-FKBP
1-min Trajectory
CFP-FRB
EMTB-CFP-FRB
Mean Speed (μm/s)
0.5
0
d
0.17
Velocity (μm/s)
0.20
0.15
0.10
0.05
0.00
CFP-FRB
EMTB-CFP-FRB
e
TGN38-mNeon-FKBP
1-min Trajectory
CFP-FRB
EMTB-CFP-FRB
Mean Speed (μm/s)
0.5
0
f
0.40
Velocity (μm/s)
0.10
0.08
0.06
0.04
0.02
0.00
CFP-FRB
EMTB-CFP-FRB
g
EMTB-CFP-FRB
1-min Trajectory
TGN38-mNeon
TGN38-mNeon-FKBP
Mean Speed (μm/s)
0.5
0
h
0.14
Velocity (μm/s)
0.12
0.10
0.08
0.06
0.04
0.02
0.00
TGN38-mNeon
TGN38-mNeon-FKBP

Figure S1 EMTB expression and FKBP tagging do not influence on vesicle mobility. a,c,e,g) COS7 cells were co-transfected with indicated mNeon- or mNeon-FKBP tagged vesicular markers (green) and CFP-FRB (blue) or EMTB-CFP-FRB (blue). The trajectories of indicated vesicles are shown. Scale bar, 10 µm. The cell boundary was highlighted by gray dotted lines. b,d,f,h) The normalized velocity of the indicated vesicular markers in CFP-FRB- or EMTB-CFP-FRB-transfected cells. *n* = 16 (CFP-FRB) and 32 cells (EMTB-CFP-FRB) in (b), 5 (CFP-FRB) and 15 cells (EMTB-CFP-FRB) in (d), 5 (CFP-FRB) and 15 cells (EMTB-CFP-FRB) in (f), 15 (TGN38-mNeon) and 25 cells (TGN38-mNeon-FKBP) in (h), from 3 independent experiments. Individual data points (black dots) and the mean ± SEM are shown. Student's t-tests were performed to generate the indicated *p*-value.

Figure S2

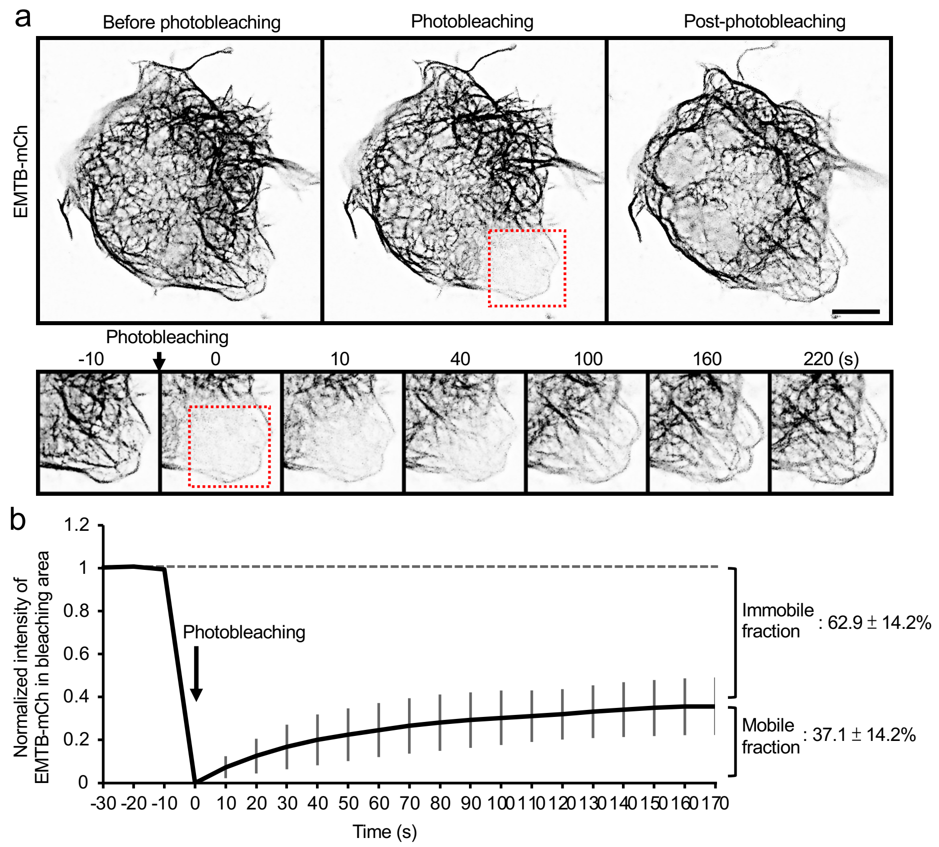

Figure S2 FRAP analysis of EMTB. a) COS7 cells transfected with EMTB-mCh were photobleached in the region of interest (red dotted square) and then allowed to recover for the indicated times. The lower panel shows the video frames of bleaching area. Scale bar, 10 μm. b) Normalized fluorescence intensity of EMTB-mCh in bleaching area in the experiment shown in (a). Data represent the mean ± SEM. *n* = 15 cells from 3 independent experiment.

Figure S3

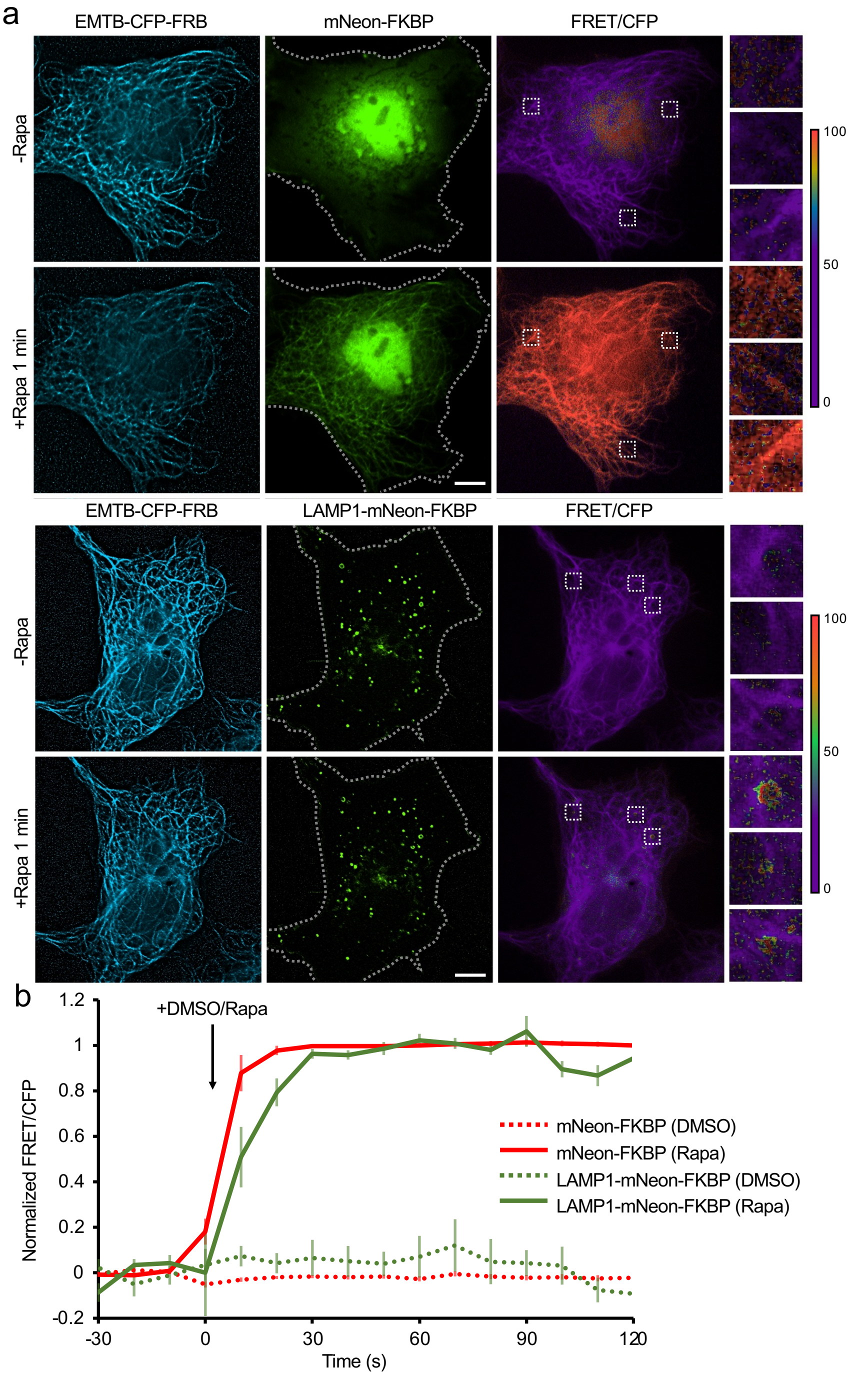

Figure S3 Inducible dimerization between MTs and target vesicles. a) COS7 cells co-transfected with EMTB-CFP-FRB (blue) and mNeon-FKBP (green, upper panel) or LAMP1-mNeon-FKBP (green, lower panel) were treated with 100 nM rapamycin (Rapa). Upon the addition of rapamycin, the mNeon-FKBP protein or FKBP-tagged lysosomes rapidly translocated onto EMTB-CFP-FRB-labeled MTs and increased the FRET signal. Enlarged views of the FRET/CFP signal are shown on the right side. Scales for the FRET/CFP intensity ratio are shown. Scale bar, 10 μm. The cell boundary was highlighted by gray dotted lines. b) The normalized level of FRET/CFP ratio in mNeon-FKBP DMSO-treated (red dotted curve; *n* = 12 cells), mNeon-FKBP rapamycin-treated (red solid curve; *n* = 7 cells), LAMP1-mNeon-FKBP DMSO-treated (green dotted curve; *n* = 6 cells) and LAMP1-mNeon-FKBP rapamycin-treated groups (green solid line; *n* = 8 cells), respectively, after the indicated time of DMSO/Rapa treatment from 3 independent experiments. Data are shown as mean ± SEM.

Figure S4

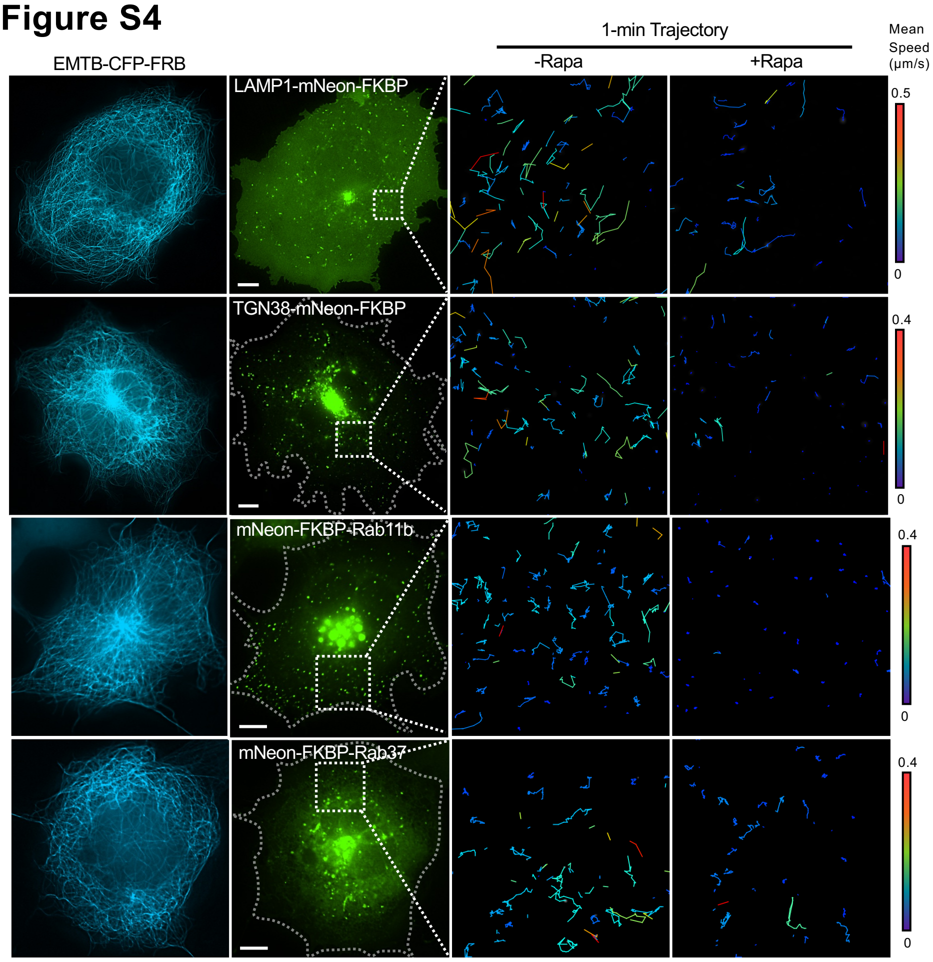

Figure S4 Rapid immobilization of intracellular trafficking by RIVET. COS7 cells co-transfected with EMTB-CFP-FRB (blue) and the indicated mNeon-FKBP tagged (green) vesicular markers were treated with 100 nM rapamycin (Rapa). The addition of rapamycin rapidly trapped mNeon-FKBP-tagged vesicles onto EMTB-CFP-FRB-labeled MTs and halted the intracellular trafficking. The enlarged mean speed trajectories of the indicated vesicular markers are shown. The cell boundary was highlighted by gray dotted lines. Scale bar, 10 μm.

Figure S5

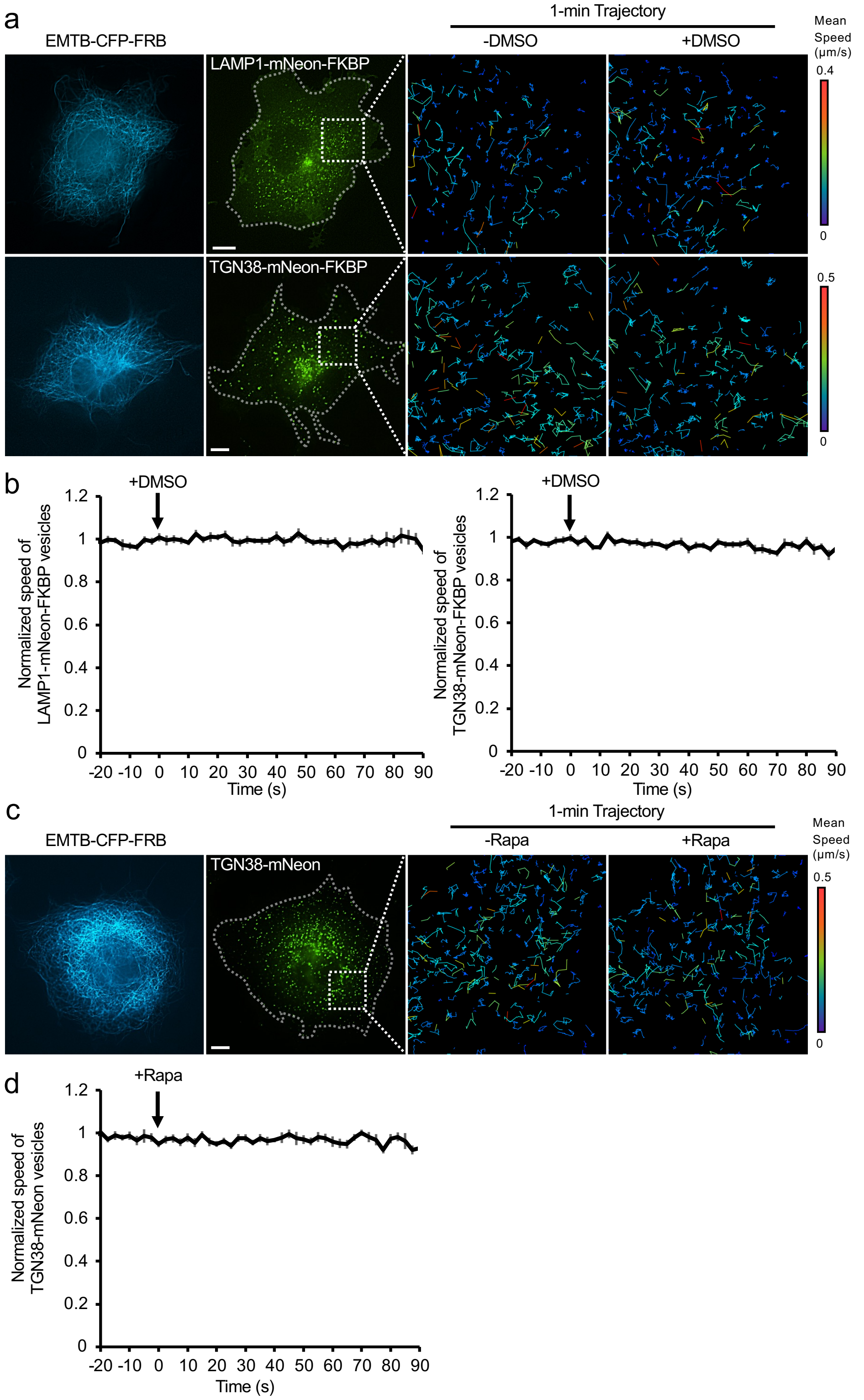

Figure S5 Vesicle motility cannot be perturbed in the absence of either chemical dimerizers or dimerizing domains tagged on VOIs. a) COS7 cells co-transfected with EMTB-CFP-FRB (blue) and the indicated mNeon-FKBP (a) or mNeon (c) tagged vesicular markers (green) were treated with 0.1% DMSO (a) or rapamycin (Rapa, 100 nM; c), respectively. The treated cells were imaged using the parameters applied in other RIVET experiments. Enlarged mean speed trajectories of the indicated VOIs are shown. Scale bar, 10 μm. b,d) The normalized mean speed of indicated vesicles upon DMSO or rapamycin treatment is shown. *n* = 19 (left, b), 25 (right, b), and 15 (d) cells from 3 independent experiments. Data are presented as mean ± SEM.

Figure S6

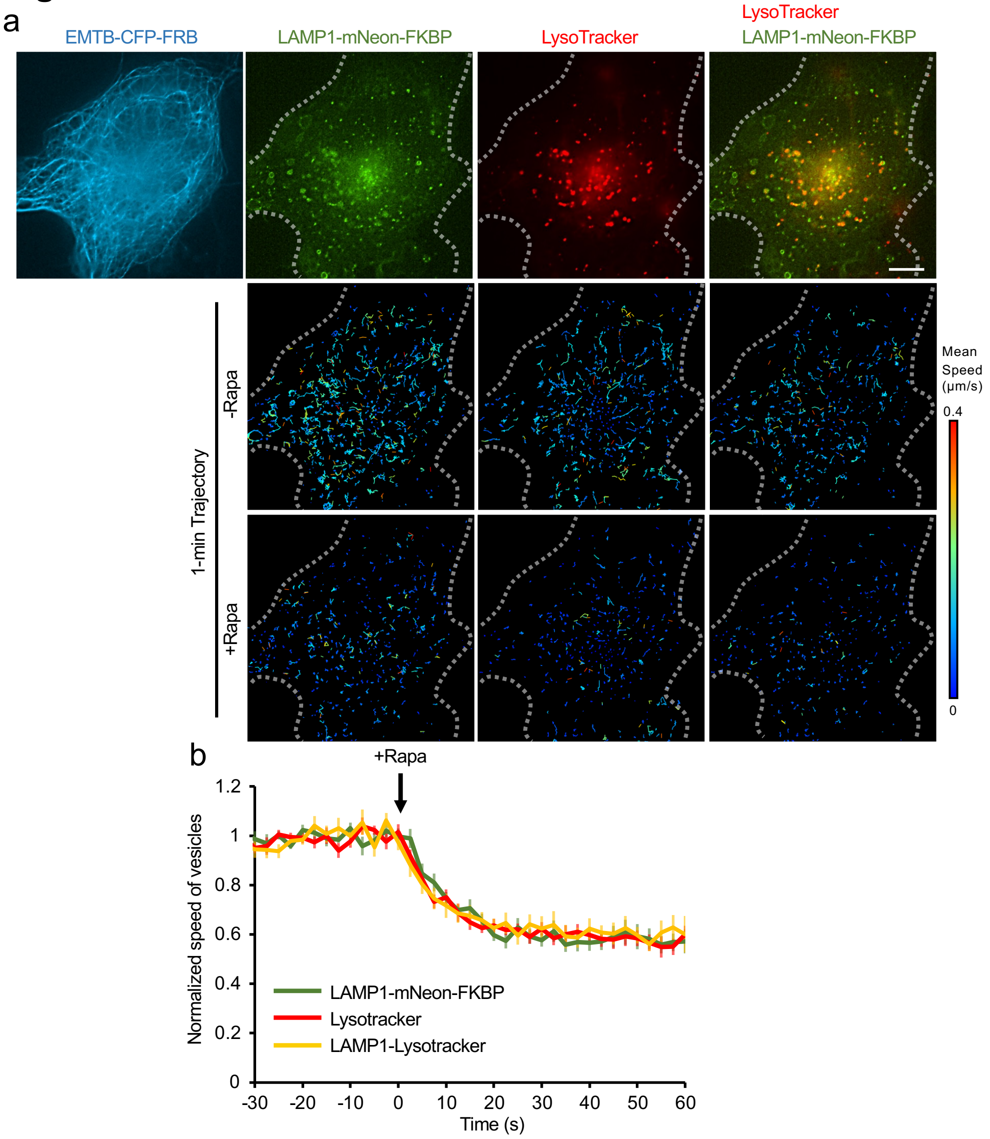

Figure S6 Immobilization of LAMP1 halts lysosome dynamics. a) COS7 cells co-transfected with EMTB-CFP-FRB (blue) and LAMP1-mNeon-FKBP (green) were stained with LysoTracker dye (red). The trajectories of LAMP1-mNeon-FKBP (left), LysoTracker red-labeled lysosomes (middle), and LAMP1/LysoTracker double-positive puncta (right) before and after 100 nM rapamycin treatment are shown. The cell boundary was highlighted by gray dotted lines. Scale bar, 10 µm. b) The normalized mean speed of LAMP1-mNeon-FKBP tagged (green curve), LysoTracker labeled (red curve) and LAMP1/Lysotracker double-positive vesicles (yellow curve) upon rapamycin treatment is shown. *n* = 10 cells from 3 independent experiments. Data are shown as mean ± SEM.

Figure S7

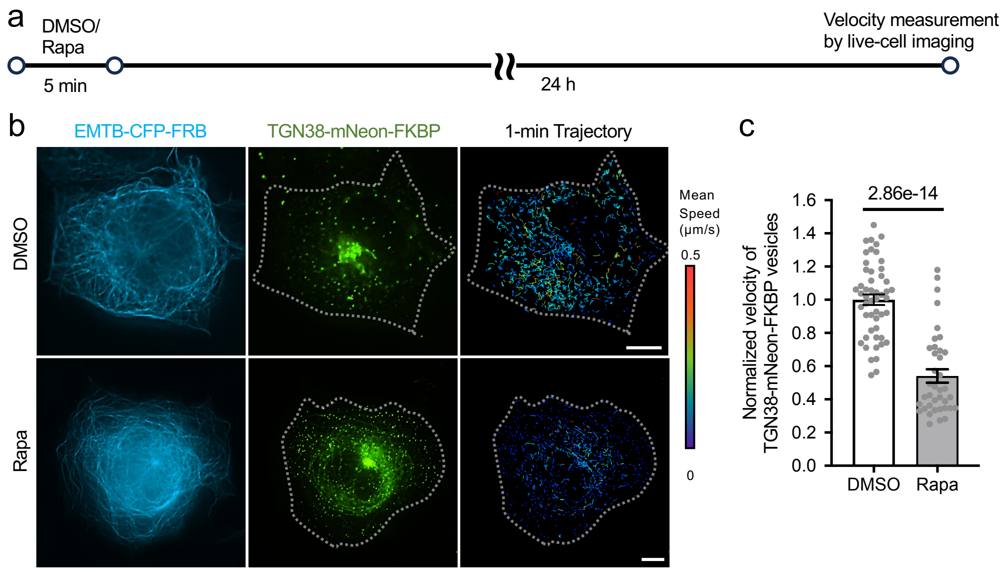

Figure S7 Long-term vesicle immobilization using RIVET. a) Cells were treated with DMSO (0.1%) or rapamycin (100 nM) for 5 min. After 5 min treatment, DMSO and rapamycin were washed out, and cells were incubated for a further 24 h followed by velocity measurement. b) COS7 cells transfected with EMTB-CFP-FRB (blue) and TGN38-mNeon-FKBP (green) were treated according to the protocol in (a). The trajectories of indicated vesicles are shown. Scale bar, 10 µm. The cell boundary was highlighted by gray dotted lines. c) Normalized velocity of the TGN38-mNeon-FKBP after transient treatment with DMSO (left) and rapamycin (Rapa, right). n = 51 (DMSO) and 38 (Rapa) cells from 3 independent experiments. Individual data points (gray dots) and the mean ± SEM are shown. Student's t-tests were performed to determine the indicated p-value.

Figure S8

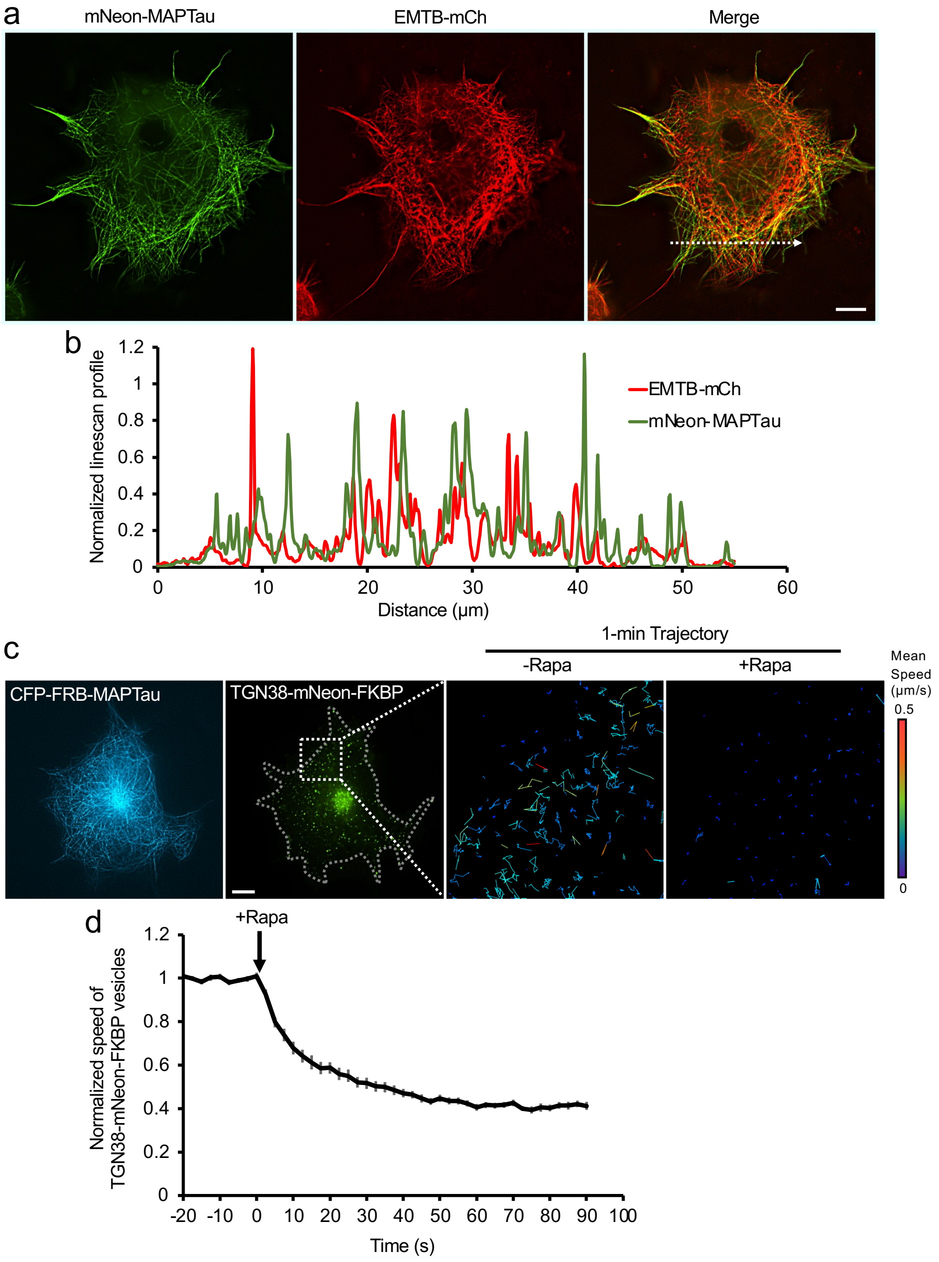

Figure S8 Using MAPTau to immobilize vesicles by RIVET. a) MAPTau and EMTB associate with different MT populations. COS7 cells were co-transfected with mNeon-MAPTau (green) and EMTB-mCh (red, mCherry, a red fluorescent protein). Scale bar, 10 µm. b) Normalized intensity profiles of EMTB-mCh (red) and mNeon-MAPTau (green) along the dotted line drawn in (a). c) COS7 cells co-transfected with CFP-FRB-MAPTau (blue) and TGN38-mNeon-FKBP (green) were treated with rapamycin (Rapa, 100 nM) for the vesicle immobilization. The enlarged mean speed trajectories of TGN38-mNeon-FKBP vesicles are shown. Scale bar, 10 µm. d) Normalized mean speed of the TGN38-mNeon-FKBP vesicles upon rapamycin (Rapa) treatment is shown. *n* = 42 cells from 3 independent experiments. Data are shown as mean ± SEM.

Figure S9

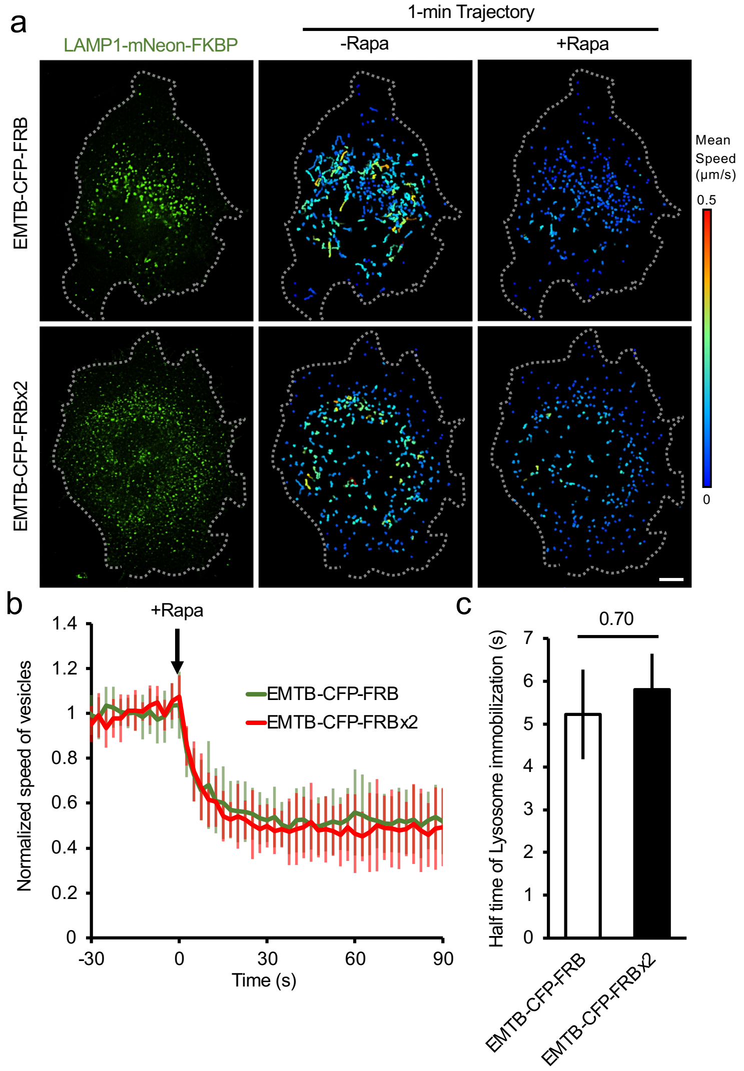

Figure S9 Trapping vesicles onto tandem FRB labeled MTs. a) COS7 cells co-transfected with LAMP1-mNeon-FKBP (green) and EMTB-CFP-FRB (upper panel) or EMTB-CFP-FRBx2 (lower panel), respectively, were treated with 100 nM rapamycin (Rapa). The trajectories of mNeon-FKBP tagged lysosomes in the EMTB-CFP-FRB and EMTB-CFP-FRBx2 groups before and after rapamycin treatment are shown. The cell boundary was highlighted by gray dotted lines. Scale bar, 10 μm. b) The normalized mean speed of lysosomes in the EMTB-CFP-FRB (green curve) and EMTB-CFP-FRBx2 (red curve) transfected cells is shown. Data are shown as mean ± SD. c) The half time of lysosome immobilization is shown. n = 16 and 9 cells in the EMTB-CFP-FRB and EMTB-CFP-FRBx2 groups, respectively, from 3 to 4 independent experiments. Data represents the mean ± SEM. Student's t-tests were performed to generate the indicated p-value.

Figure S10

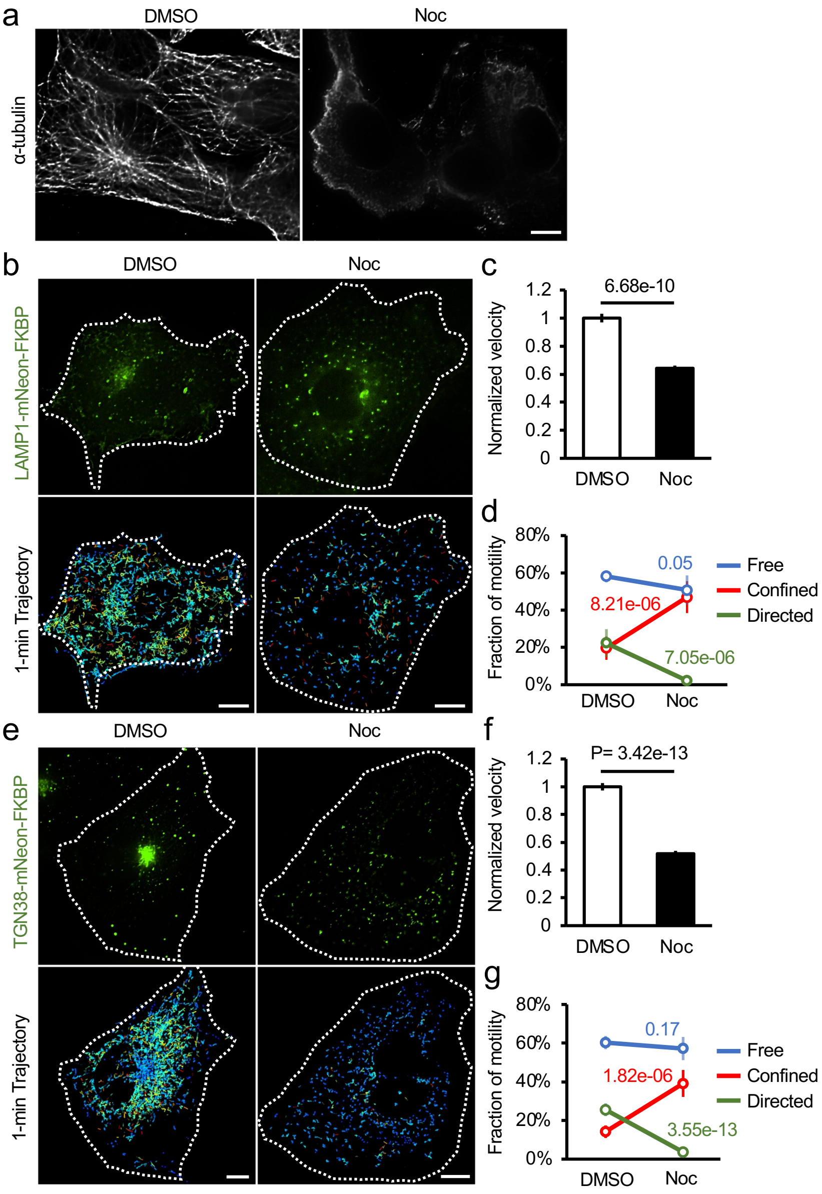

Figure S10 The effects of MT removal on vesicular trafficking. a) COS7 cells treated with 0.1% DMSO or 3.3 μM Nocodazole (Noc) for 2 h were immunostained with α-tubulin to label MTs. b,e) COS7 cells transfected with LAMP1-mNeon-FKBP (b) or TGN38-mNeon-FKBP (e) were treated with 0.1% DMSO or 3.3 μM Nocodazole (Noc) for 2 h. The trajectories of lysosomes and post-Golgi vesicles in different conditions are shown. The cell boundary was highlighted by white dotted lines. Scale bar, 10 μm. c,f) The normalized velocities of lysosomes (c) and post-Golgi vesicles (f) in the indicated conditions. *n* = 13 (DMSO) and 13 cells (Noc) in (c), 13 (DMSO) and 13 cells (Noc) in (f), from 4 independent experiments. Data are shown as mean ± SEM. d,g) The motility type of lysosomes (d) and post-Golgi vesicles (g) changed after 2 h of nocodazole treatment. *n* = 8 (DMSO) and 8 cells (Noc) in (d), 13 (DMSO) and 13 cells (Noc) in (g), from 3 independent experiments. Data are shown as mean ± SD. Student's *t*-tests were performed, with the resulting *p*-values indicated.

Figure S11

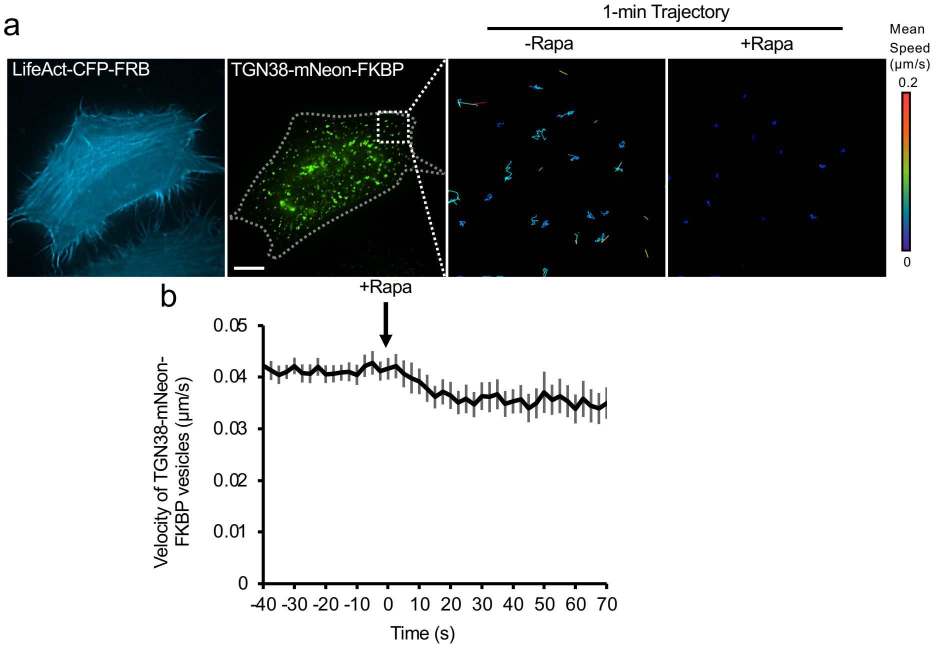

Figure S11 Trapping vesicles on actin filaments after MT depolymerization. a) COS7 cells co-transfected with LifeAct-CFP-FRB (blue) and TGN38-mNeon-FKBP (green) were pretreated with nocodazole (3.3 μM) for 2 h for MT disassembly. After that, rapamycin (Rapa, 100 nM) was treated to trigger dimerization between LifeAct-CFP-FRB and TGN38-mNeon-FKBP. The enlarged mean speed trajectories of indicated vesicular markers are shown. Scale bars, 10 μm. b) The normalized mean speed of the indicated vesicles upon rapamycin treatment is shown. *n* = 19 cells from 3 independent experiments. Data are shown as mean ± SEM.

Figure S12

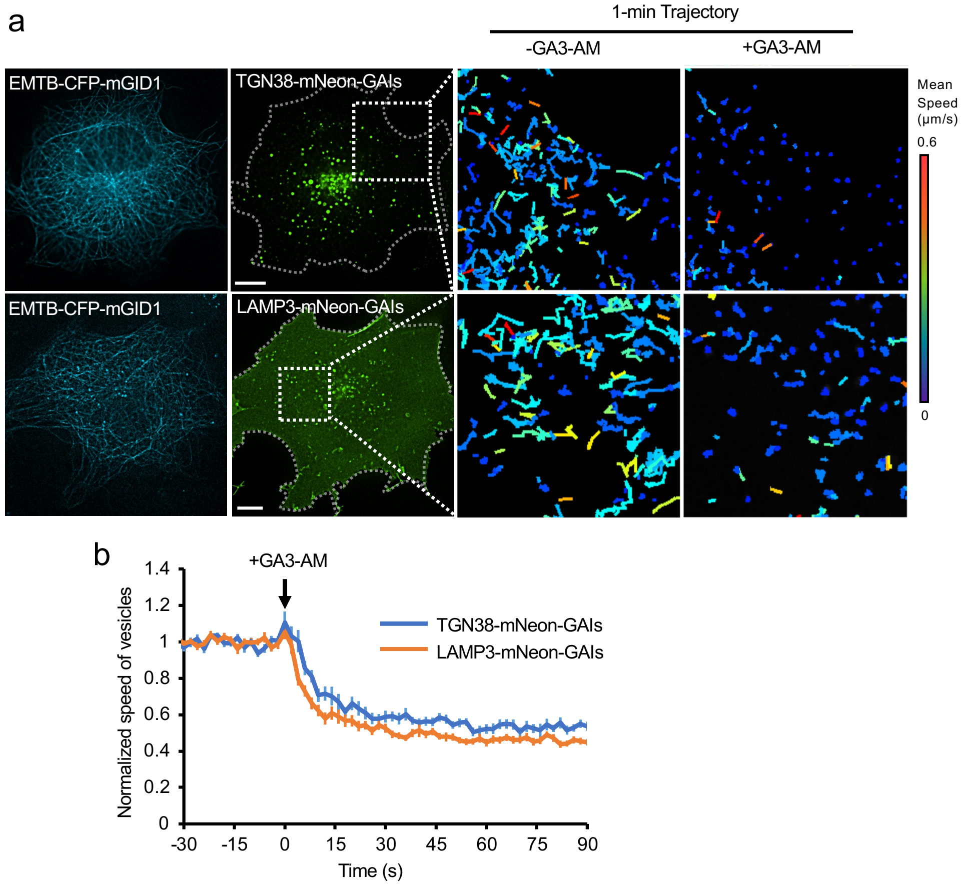

Figure S12 Triggering RIVET by gibberellin system. a) COS7 cells co-transfected with EMTB-CFP-mGID1 (blue) and TGN38-mNeon-GAIs (green; upper panel) or LAMP3-mNeon-GAIs (green; lower panel), respectively, were treated with 100 μM GA3-AM. The enlarged mean speed trajectories of the indicated vesicular markers are shown. The cell boundary was highlighted by gray dotted lines. Scale bar, 10 μm. b) The normalized mean speed of the indicated vesicles in (a) is shown. *n* = 16 and 17 cells in the LAMP3-mNeon-GAIs (orange curve) and TGN38-mNeon-GAIs (blue curve) groups, respectively, from 3 independent experiments. Data are shown as mean ± SEM.

Figure S13

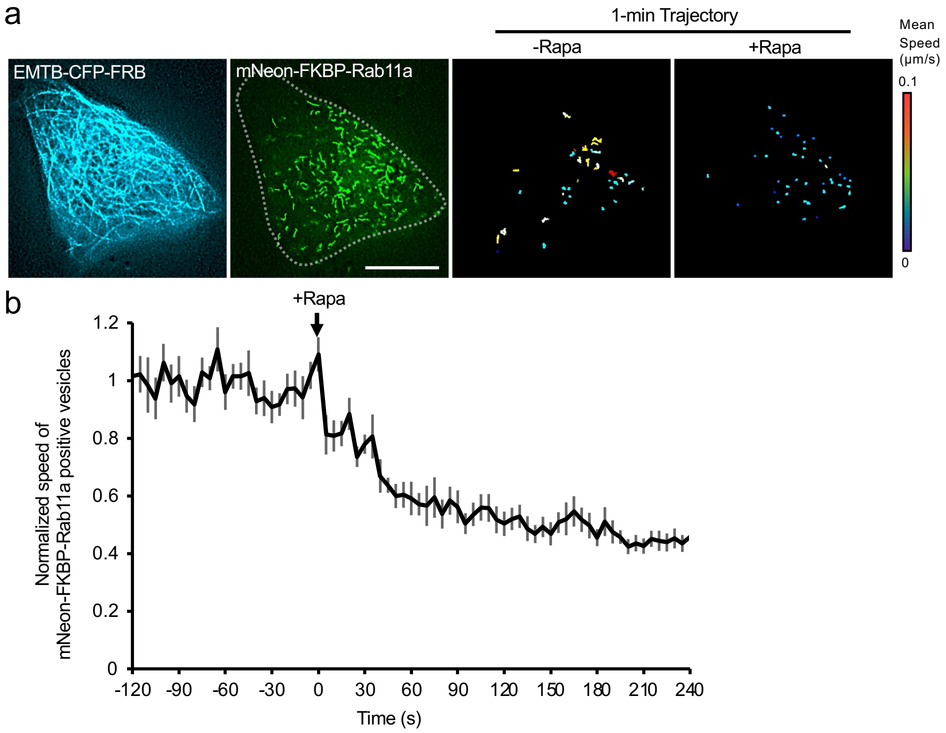

Figure S13 Immobilization of recycling endosomes in MDCK cells. a) MDCK cells co-transfected with EMTB-CFP-FRB (blue) and mNeon-FKBP-Rab11a (green) were treated with 100 nM rapamycin (Rapa). The mean speed trajectories upon rapamycin treatment are shown. The cell boundary was highlighted by gray dotted lines. Scale bar, 10 µm. b) The normalized mean speed of mNeon-FKBP-Rab11a is shown. *n* = 11 cells from 3 independent experiments. Data are shown as mean ± SEM.

Figure S14

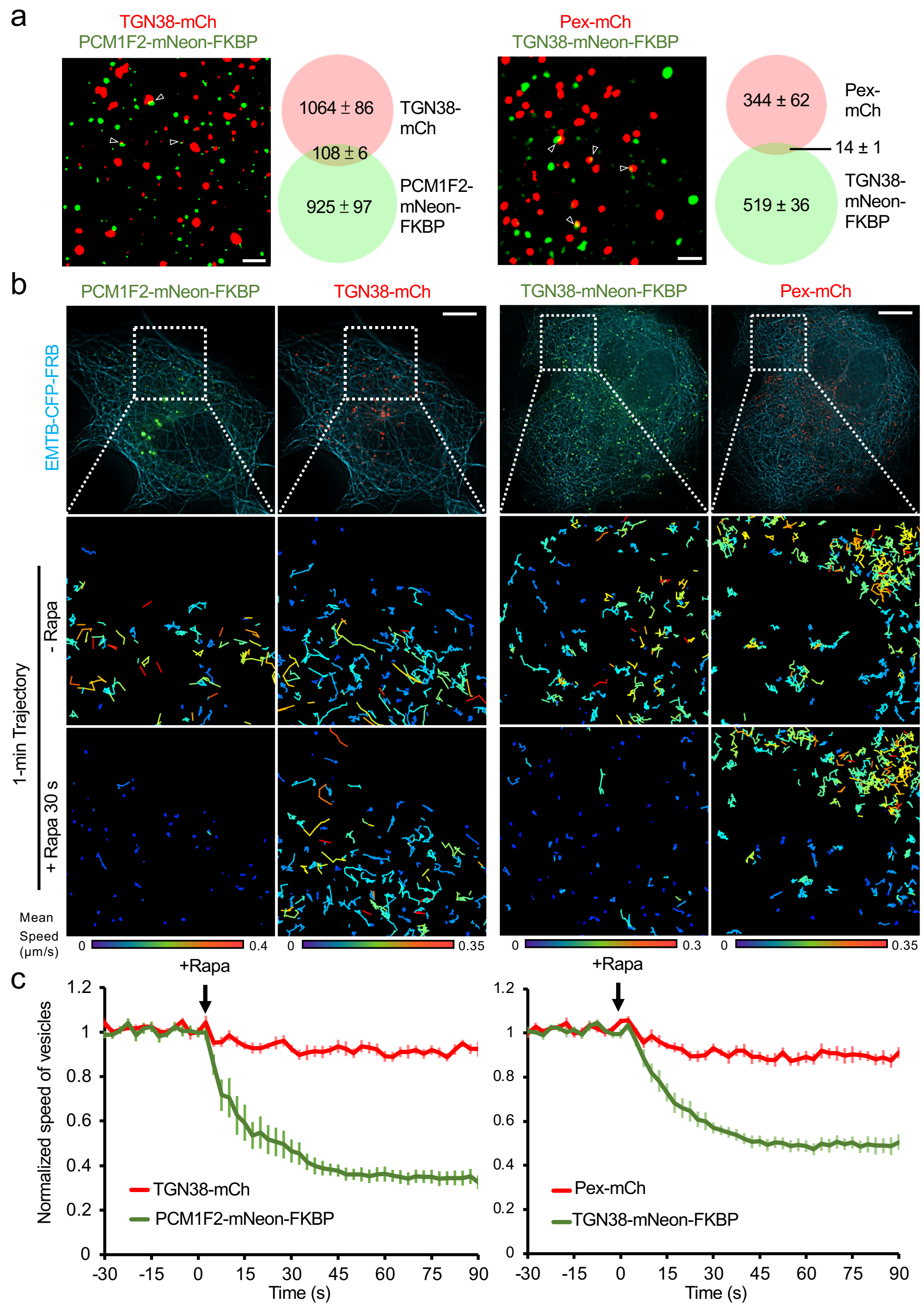

Figure S14 Using RIVET to specifically immobilize target vesicles without affecting unrelated vesicle motility. a) The fluorescence images show the distribution of the indicated vesicles in COS7 cells. Vesicles with overlapped markers are indicated by arrowheads. Scale bar, 2 μm. The number of overlapping and non-overlapping vesicles in two combinations are showed as Venn diagrams. Data are shown as mean ± SEM. *n* = 5 cells in each condition. b) COS7 cells transfected with the indicated constructs were treated with 100 nM rapamycin (Rapa). The trajectories of the indicated vesicles before and after 100 nM rapamycin treatment are shown. Scale bar, 10 μm. c) The normalized mean speed of the indicated vesicles as shown in (b). *n* = 12 (left) and 10 (right) cells from 3 to 4 independent experiments. Data are shown as mean ± SEM.

Figure S15

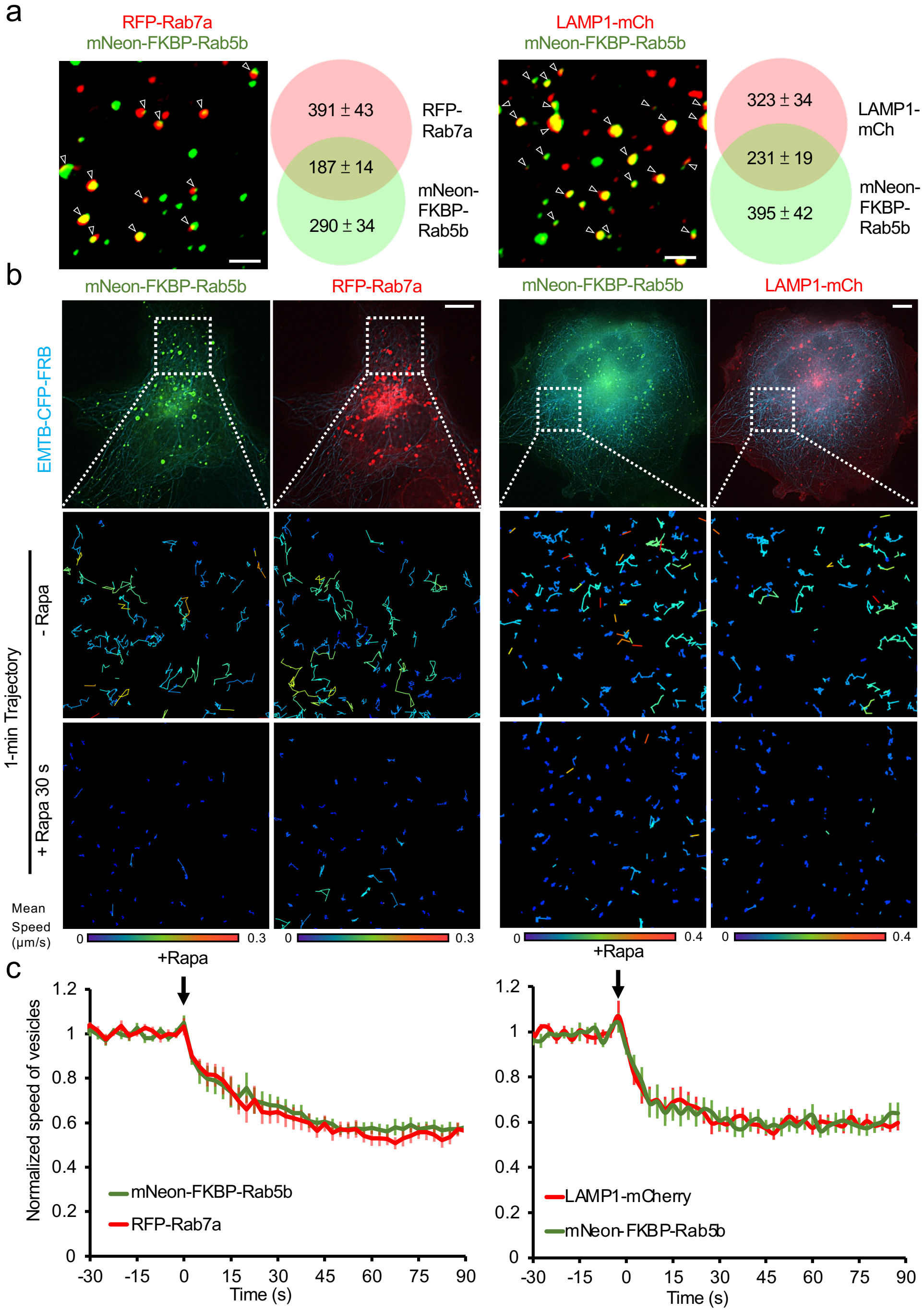

Figure S15 RIVET simultaneously halts target vesicles and their associated vesicles. a) The fluorescence images show the distribution of the indicated vesicles in COS7 cells. Vesicles with overlapped markers are indicated by arrowheads. Scale bar, 2 µm. The number of overlapping and non-overlapping vesicles in two combinations are showed as Venn diagrams. Data are shown as mean ± SEM. *n* = 5 cells in each condition. b) COS7 cells transfected with the indicated constructs were treated with 100 nM rapamycin (Rapa). The trajectories of indicated vesicles before and after 100 nM rapamycin treatment are shown. Scale bar, 10 µm. c) The normalized mean speed of the indicated vesicles shown in (b). *n* = 13 and 9 cells from 3 independent experiments. Data are shown as mean ± SEM.

Figure S16

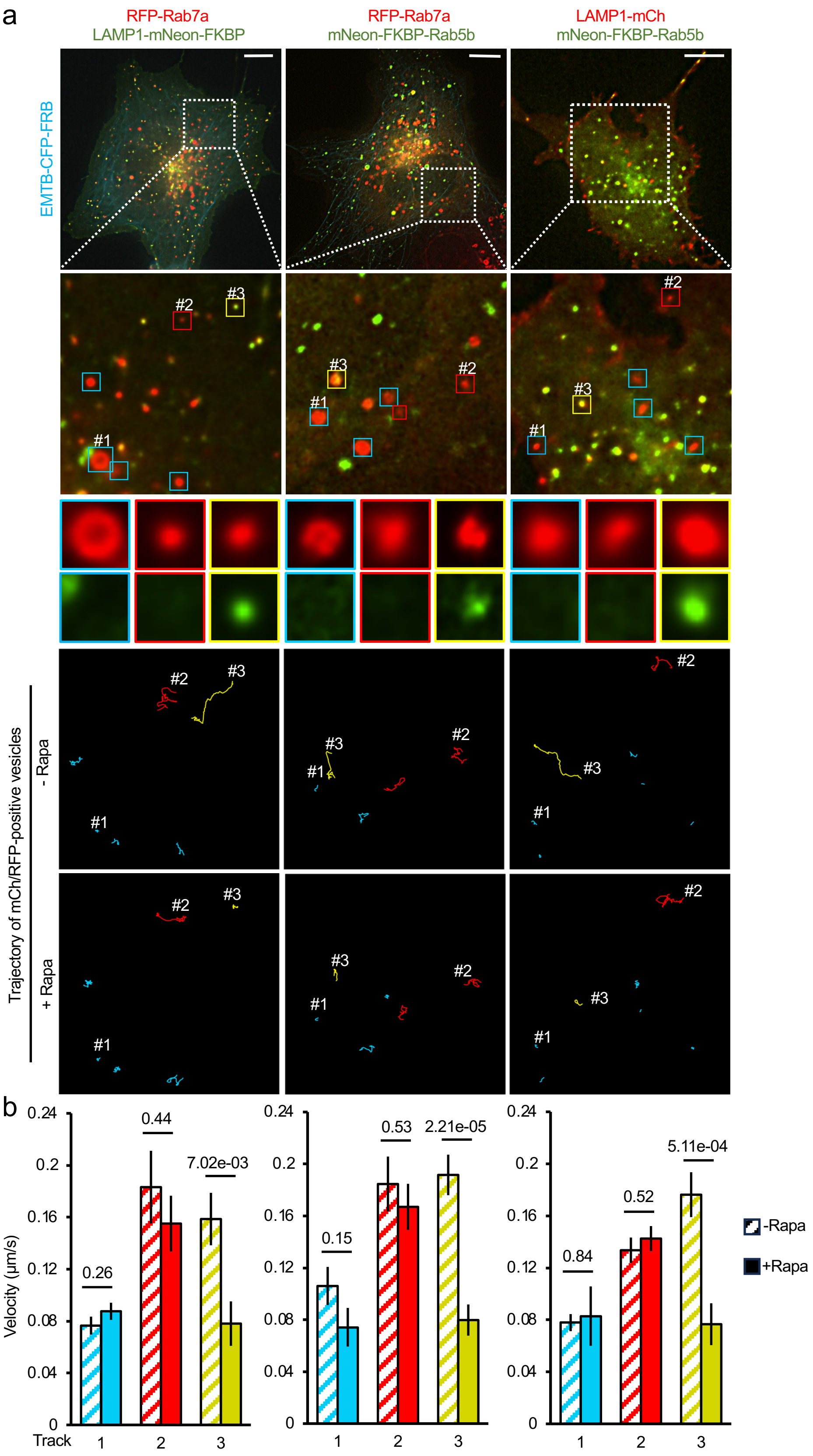

a
RFP-Rab7a
LAMP1-mNeon-FKBP
RFP-Rab7a
mNeon-FKBP-Rab5b
LAMP1-mCh
mNeon-FKBP-Rab5b
EMTB-CFP-FRB
#1
#2
#3
Trajectory of mCh/RFP-positive vesicles
- Rapa
+ Rapa
b
Velocity (μm/s)
0.24
0.2
0.16
0.12
0.08
0.04
0
0.26
0.44
7.02e-03
0.15
0.53
2.21e-05
0.84
0.52
5.11e-04
Track
1
2
3
-Rapa
+Rapa

Figure S16 RIVET specifically halts FKBP-tagged vesicles and their overlapping vesicles. a) COS7 cells co-transfected with EMTB-CFP-FRB (blue) and three combinations of vesicles (green and red) were treated with 100 nM rapamycin (Rapa). Based on vesicle markers and motility types, we identified three types of RFP/mCh-positive vesicles: 1) Confined FKBP-negative vesicles (blue squares and tracks); 2) Highly dynamic FKBP-negative vesicles (red squares and tracks); and 3) FKBP-positive vesicles (yellow squares and tracks). Representative trajectories of these vesicles before and after rapamycin treatment are shown. Scale bar: 10 µm. b) The velocity of the defined vesicle types before and after rapamycin treatment is presented. n = 60 (RFP-Rab7a/LAMP1-mNeon-FKBP), 60 (RFP-Rab7a/mNeon-FKBP-Rab5b), and 60 (LAMP1-mCh/mNeon-FKBP-Rab5b) tracks from 3 independent experiments. Data represent the mean ± SEM. Student's t-tests were used to calculate the indicated p-values. The trajectories of mCh/RFP-positive vesicles show that only the movement of FKBP-positive vesicles (track #3) was disrupted by RIVET, while the highly dynamic (track #2) and confined (track #1) non-FKBP-tagged vesicles were unaffected following rapamycin treatment.

Figure S17

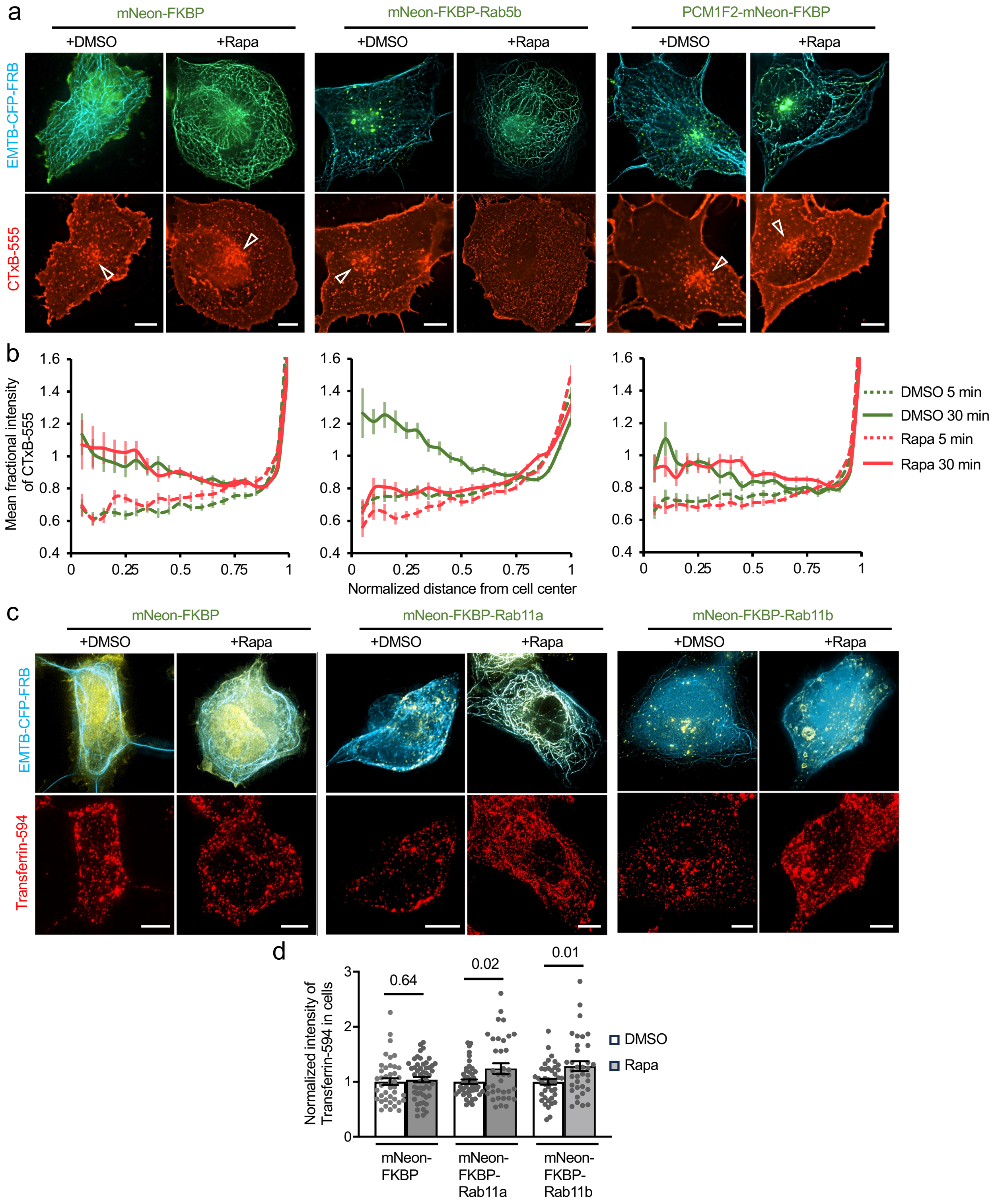

a
mNeon-FKBP
+DMSO
+Rapa
mNeon-FKBP-Rab5b
+DMSO
+Rapa
PCM1F2-mNeon-FKBP
+DMSO
+Rapa
EMTB-CFP-FRB
CTxB-555
b
Mean fractional intensity of CTxB-555
Normalized distance from cell center
DMSO 5 min
DMSO 30 min
Rapa 5 min
Rapa 30 min
c
mNeon-FKBP
+DMSO
+Rapa
mNeon-FKBP-Rab11a
+DMSO
+Rapa
mNeon-FKBP-Rab11b
+DMSO
+Rapa
EMTB-CFP-FRB
Transferrin-594
d
Normalized intensity of Transferrin-594 in cells
0.64
0.02
0.01
DMSO
Rapa
mNeon-FKBP
mNeon-FKBP-Rab11a
mNeon-FKBP-Rab11b

Figure S17 RIVET specifically inhibits vesicular trafficking and corresponding cargo delivery. a) COS7 cells co-transfected with EMTB-CFP-FBB (blue) and mNeon-FKBP, mNeon-FKBP-Rab5b, PCM1F2-mNeon-FKBP (green), respectively, were treated with 0.1 % DMSO or 100 nM rapamycin (Rapa). Treated cells were then incubated with CTxB-Alexa Fluor 555 (red) for 30 min. Accumulation of CTxB-Alexa Fluor 555 in the perinuclear regions of the cells was highlighted by arrowheads. Scale bar, 10 µm. b) The mean fractional intensity of CTxB-Alexa Fluor 555 distributed from the cell center to the cell periphery in the indicated conditions is shown. n = 159 (mNeon-FKBP), 277 (mNeon-FKBP-Rab5b), and 181 cells (PCM1F2-mNeon-FKBP) from 3 to 4 independent experiments. Data are shown as mean ± SEM. c) COS7 cells co-transfected with the indicated constructs were serum-starved for 3 h and subsequently treated with 0.1 % DMSO or 100 nM rapamycin (Rapa). Cells were then incubated with Alexa Fluor 594-conjugated transferrin (red). After washing out excessive transferrin-Alexa Fluor 594, cells were then incubated in serum-containing medium at 37˚C for 30 min and imaged. Scale bar, 10 µm. d) The normalized intensity of transferrin-Alexa Fluor 594 in (c). n = 41, 52, 48, 36, 40, and 35 cells from left to right; 3 independent experiments. Individual data points (gray) and the mean ± SD (black) are shown. Student's t-tests were performed, with the resulting p-values indicated.

Figure S18

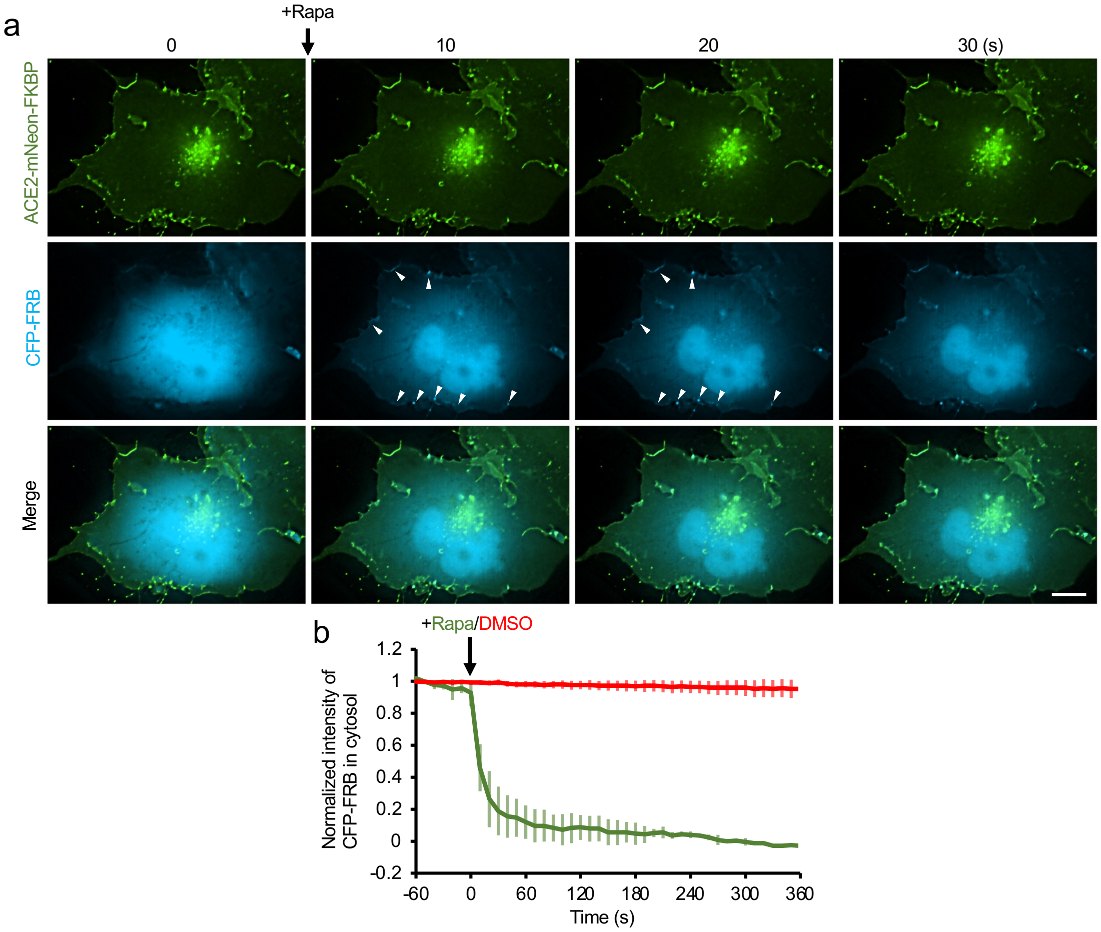

Figure S18 Rapidly triggering dimerization on ACE2-FKBP-labeled components on plasma membrane. a) COS7 cells co-transfected with ACE2-mNeon-FKBP (green) and CFP-FRB (blue) were treated with rapamycin (Rapa, 100 nM). The translocation of CFP-FRB from cytosol onto ACE2-FKBP-labeled plasma membrane (arrowheads). Scale bar, 10 µm. b) Normalized intensity of CFP-FRB in the cytosol of cells in (a) upon 0.1% DMSO (red curve) or 100 nM rapamycin (Rapa, green curve) treatment is shown. *n* = 17 and 22 cells in the DMSO and rapamycin groups, respectively, from 3 independent experiments. Data are shown as mean ± SD.

Figure S19

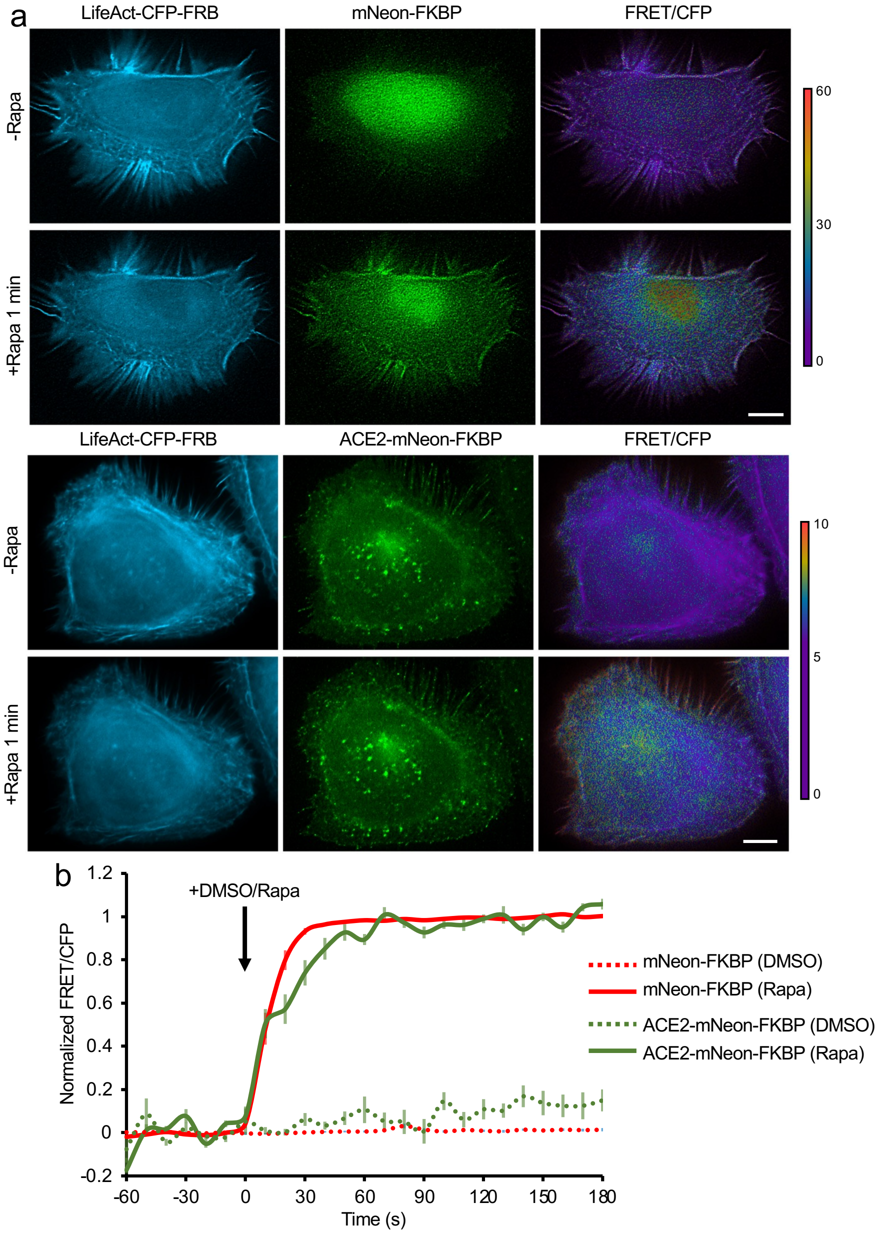

Figure S19 Rapid dimerization between actin filaments and target components. a) COS7 cells co-transfected with LifeAct-CFP-FRB and mNeon-FKBP (upper panel) and ACE2-mNeon-FKBP (lower panel), respectively, were treated with rapamycin (Rapa, 100 nM). The addition of rapamycin rapidly trapped mNeon-FKBP or ACE2-mNeon-FKBP onto LifeAct-FRB-labeled actin filaments, resulting in an increased FRET signal. Scales for the FRET/CFP intensity ratio are shown. Scale bar, 10 µm. b) The normalized level of FRET/CFP ratio in cells transfected with the indicated constructs upon 0.1% DMSO or 100 nM rapamycin treatment is shown. *n* = 15 (mNeon-FKBP) and 17 cells (ACE2-mNeon-FKBP) from 3 independent experiments. Data are shown as mean ± SEM.

Figure S20

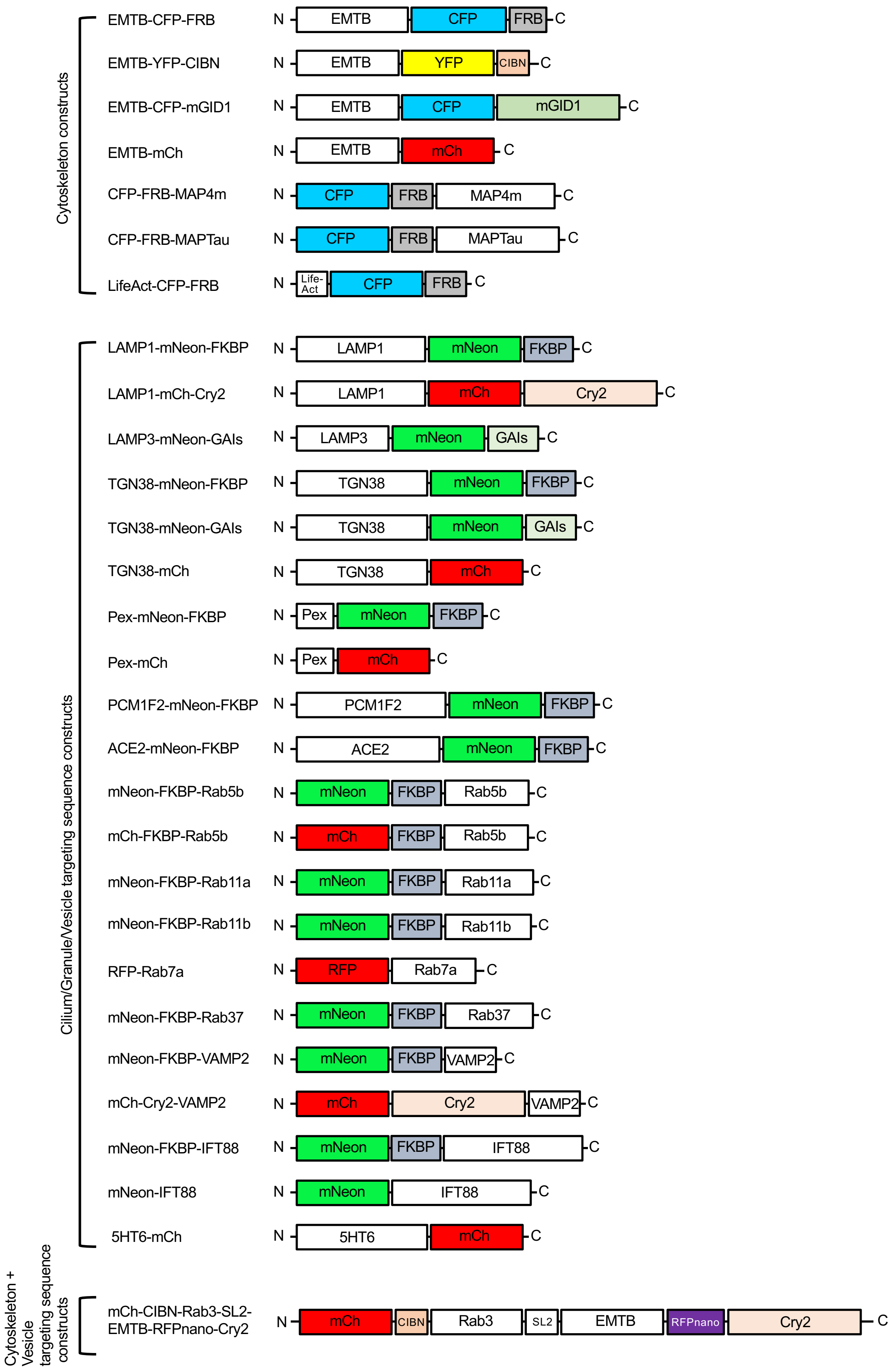

Figure S20 The design of constructs used in this study.

Video S1
FKBP tagging does not affect vesicle motility. COS7 cells co-transfected with EMTB-CFP-FRB (blue) and either TGN38-mNeon (green, upper panel) or TGN38-mNeon-FKBP (green, lower panel) were imaged every 2.5 s for 85 s. Scale bar, 10 µm. See also Figure S1g, Supporting Information.

Video S2
FRAP analysis of EMTB. COS7 cells transfected with EMTB-mCh were photobleached locally (red square) and then allowed to recover for the indicated times. Images were taken every 10 s for 230 s. Scale bar, 10 µm. See also Figure S2a, Supporting Information.

Video S3
RIVET rapidly induces dimerization between vesicles and MTs. COS7 cells co-transfected with EMTB-CFP-FRB (blue) and either mNeon-FKBP (upper panel; green) or LAMP1-mNeon-FKBP (lower panel; green) were treated with rapamycin (100 nM). FRET-based images were captured every 10 s for a duration of 180 s. Scale bar, 10 µm. See also Figure S3a, Supporting Information.

Video S4
Rapid immobilization of intracellular trafficking using RIVET. COS7 cells co-transfected EMTB-CFP-FRB (blue) and mNeon-FKBP (NF)-tagged vesicular markers (green) were treated with rapamycin (100 nM). Treated cells were imaged every 2.5 s for 3 min. Scale bar, 10 µm. See also Figure 1b and Figure S4, Supporting Information.

Video S5
Vesicle motility cannot be perturbed in the absence of either chemical dimerizers or dimerizing domains tagged on VOIs. COS7 cells co-transfected with EMTB-CFP-FRB (blue) and the indicated mNeon-FKBP or mNeon tagged vesicular markers (green) were treated with 0.1% DMSO or rapamycin (Rapa, 100 nM), respectively. The treated cells were imaged every 2.5 s for 160 s using the parameters applied in other RIVET experiments. Scale bar, 10 µm. See also Figure S5a,c, Supporting Information.

Video S6
Immobilization of LAMP1 halts lysosome dynamics. COS7 cells co-transfected with EMTB-CFP-FRB and LAMP1-mNeon-FKBP (green) were stained with LysoTracker dye (red). The cells were treated with rapamycin (Rapa, 100 nM) and imaged every 2.5 s for 145 s. Scale bar, 10 µm. See also Figure S6, Supporting Information.

Video S7
Long-term vesicle immobilization using RIVET. Cells were treated with DMSO (0.1%) or rapamycin (100 nM) for 5 min. After 5 min treatment, DMSO and rapamycin were washed out, and cells were incubated for further 24 h following by velocity measurement. COS7 cells transfected with EMTB-CFP-FRB (blue) and TGN38-mNeon-FKBP (green) were treated according to aforementioned protocol. Cells were imaged every 2.5 s for 90 s. Scale bar, 10 µm. See also Figure S7b, Supporting Information.

Video S8
Using MAPTau to immobilize vesicles by RIVET. COS7 cells co-transfected with CFP-FRB-MAPTau (blue) and TGN38-mNeon-FKBP (green) were treated with rapamycin (Rapa, 100 nM) for the vesicle immobilization. The cells were imaged every 2.5 s for 175 s. Scale bar, 10 µm. See also Figure S8c, Supporting Information.

Video S9
Vesicle immobilization on MTs labeled by different copy of dimerization domains. COS7 cells co-transfected with LAMP1-mNeon-FKBP (green) and either EMTB-CFP-FRB (blue; upper panel) or tandem FRB, EMTB-CFP-FRBx2 (blue; lower panel) were treated with 100 nM rapamycin (Rapa). Treated cells were imaged every 2.5 s for a period of 12.5 min. Scale bar, 10 µm. See also Figure S9a, Supporting Information.

Video S10
The effect of MT removal on vesicle motility. COS7 cells transfected with mNeon-FKBP-tagged vesicular markers, either LAMP1 (green; upper panel) or TGN38-mNeon-FKBP (green; lower panel) were treated with DMSO (0.1 %; left panel) and nocodazole (Noc; 3.3 µM; right panel) for MT disassembly. Images were taken every 2.5 s for 117.5 s. Scale bar, 10 µm. See also Figure S10b,e, Supporting Information.

Video S11

Trapping vesicles on actin filaments after MT depolymerization. COS7 cells co-transfected with LifeAct-CFP-FRB (blue) and TGN38-mNeon-FKBP (green) were pretreated with nocodazole (3.3 µM) for 2 h for MT disassembly. After that, rapamycin (Rapa, 100 nM) was treated to trigger dimerization between LifeAct-CFP-FRB and TGN38-mNeon-FKBP. Images were taken every 2.5 s for 167.5 s. Scale bars, 10 µm. See also Figure S11a, Supporting Information.

Video S12

Using gibberellin system to immobilize post-Golgi vesicles. COS7 cells co-transfected with EMTB-CFP-mGID1 and TGN38-mNeon-GAIs (green) were treated with the engineering gibberellin, GA3-AM (100 µM) to trap the indicated vesicles on MTs. Images were taken every 2.5 s for 5 min. Scale bar, 10 µm. See also Figure S12a, upper panel, Supporting Information.

Video S13

Using gibberellin system to immobilize lysosomes. COS7 cells co-transfected with EMTB-CFP-mGID1 and LAMP3-mNeon-GAIs (green) were treated with the engineering gibberellin, GA3-AM (100 µM) to trap the indicated vesicles onto MTs. Images were taken every 2.5 s for 5 min. Scale bar, 10 µm. See also Figure S12a lower panel, Supporting Information.

Video S14

RIVET halts recycling endosomes in MDCK kidney cells. MDCK kidney cells co-transfected with EMTB-CFP-FRB (blue) and mNeon-FKBP-Rab11a (green) were treated with rapamycin (100 nM) to halt the vesicle movement. Images were taken every 5 s for 10 min. Scale bar, 10 µm. See also Figure S13a ,Supporting Information.

Video S15

Immobilization of FKBP-tagged centriolar satellites does not affect peroxisome motility. COS7 cells co-transfected with EMTB-CFP-FRB, PCM1F2-mNeon-FKBP (green), and Pex-mCh (red) were treated with 100 nM rapamycin. Images were taken every 2.5 s for 3 min. Scale bars are shown. See also Figure 2b.

Video S16

Immobilization of FKBP-tagged centriolar satellites does not affect post-Golgi vesicle motility. COS7 cells co-transfected with EMTB-CFP-FRB, PCM1F2-mNeon-FKBP (green), and TGN38-mCh (red) were treated with 100 nM rapamycin. Images were taken every 2.5 s for 3 min. Scale bars are shown. See also Figure S14b, left panel, Supporting Information.

Video S17

Immobilization of FKBP-tagged post-Golgi vesicles does not affect peroxisome motility. COS7 cells co-transfected with EMTB-CFP-FRB, TGN38-mNeon-FKBP (green), and Pex-mCh (red) were treated with 100 nM rapamycin. Images were taken every 2.5 s for 3 min. Scale bars are shown. See also Figure S14b, right panel, Supporting Information.

Video S18

Immobilization of FKBP-tagged lysosomes simultaneously suppresses Rab7a-positive vesicle motility. COS7 cells co-transfected with EMTB-CFP-FRB, LAMP1-mNeon-FKBP (green) and RFP-Rab7a (red) were treated with 100 nM rapamycin. Images were taken every 2.5 s for 3 min. Scale bars are shown. See also Figure 2e.

Video S19

Immobilization of FKBP-tagged Rab5b vesicles simultaneously suppresses Rab7a vesicle motility. COS7 cells co-transfected with EMTB-CFP-FRB, mNeon-FKBP-Rab5b (green) and RFP-Rab7a (red) were treated with 100 nM rapamycin. Images were taken every 2.5 s for 3 min. Scale bars are shown. See also Figure S15b, left panel, Supporting Information.

Video S20

Immobilization of FKBP-tagged Rab5b vesicles simultaneously suppresses the lysosome motility. COS7 cells co-transfected with EMTB-CFP-FRB, mNeon-FKBP-Rab5b (green) and LAMP1-mCh (red) were treated with 100 nM rapamycin. Images were taken every 2.5 s for 3 min. Scale bars are shown. See also Figure S15b ,right panel, Supporting Information.

Video S21

Using optogenetic RIVET to rapidly immobilize lysosomes in a reversible manner. COS7 cells co-transfected with EMTB-YFP-CIBN and LAMP1-mCh-Cry2 (red) were illuminated by blue light for 3 min. Images were taken at a 5 s interval for 20 min. Scale bar, 10 µm. See also Figure 3a.

Video S22
Locally immobilizing lysosomes using optogenetic RIVET. COS7 cells co-transfected with EMTB-YFP-CIBN and LAMP1-mCh-Cry2 (red) were illuminated by blue light in a specific region (a dotted circle). The enlarged images in the light-illuminated regions and three non-light illuminated regions (dotted squares) are shown. Images were taken at a 5 s interval for 10 min. Scale bar, 10 μm for whole cell and 2 μm for enlarged view. See also Figure 3c.

Video S23
Rapid immobilization of IFT by RIVET. NIH3T3 fibroblasts co-transfected with CFP-FRB-MAP4m, mNeon-IFT88 (Left) and mNeon-FKBP-IFT88 (Right), respectively, were serum-starved for 24 hr to induce ciliogenesis. The ciliated cells were then treated with rapamycin (Rapa, 100 nM) and imaging every 200 ms for 40 s. Scale bar, 5 μm. See also Figure 4b.

Video S24
Acute IFT immobilization disrupts ciliary structure. NIH3T3 fibroblasts co-transfected with CFP-FRB-MAP4m (blue), mNeon-FKBP-IFT88 (green), and 5HT6-mCh (red) were serum-starved for 24 h to induce ciliogenesis. The ciliated cells were then treated with DMSO (0.1%; upper panel) or rapamycin (Rapa, 100 nM; lower panel) and imaging every 5 min for 245 min. Scale bar, 5 μm. See also Figure 4d.

Video S25
Rapid immobilization of synaptic vesicles by RIVET in primary cultured cortical neurons. Primary cultured cortical neurons co-expressed EMTB-CFP-FRB (blue) and mNeon-FKBP-VAMP2 (green) were treated with 100 nM rapamycin (Rapa). The trajectories of mNeon-FKBP-VAMP2 in three indicated regions are shown. Images were taken every 10 s for 590 s. Scale bars, 10 μm. See also Figure 5c.

Video S26
Using optogenetic RIVET to immobilize synaptic vesicles in a reversible manner. COS7 cells co-transfected with EMTB-YFP-CIBN and mCh-Cry2-VAMP2 (red) were illuminated by blue light for 3 min. The trajectories of mCh-Cry2-VAMP2 in the indicated region are shown. Images were taken every 5 s for 20 min. Scale bar, 10 μm for whole cell and 2 μm for enlarged view. See also Figure 5e.

Video S27
Immobilization of synaptic vesicles on MTs in *C. elegans*. The transgenic *C. elegans* crying pHSP16/2::mCh-CIBN-Rab3-SL2-EMTB-RFPnano-Cry2 and pUNC-104::GFP were stimulated by blue light. The dynamics of mCh-CIBN-Rab3 before and after 3-min of blue light stimulation (488 nm; 6.9 mW/mm2) was monitored by light-cell imaging. Images were taken every 200 ms for 99 s. Scale bar, 10 μm. See also Figure 6a.

Video S28
Immobilization of synaptic vesicles attenuates swimming of *C. elegans*. Wide type *C. elegans* (left) and transgenic C. elegans co-expressing EMTB-Cry2 and CIBN-Rab3 (right) were placed in M9 buffer and were temporarily illuminated by blue light (488 nm; 6.9 mW/mm2) for 7 min. Images were taken every 50 ms for 9.73 min. See also Figure 6b.

Video S29
Immobilization of ACE2-positive vesicles on cortical actin blocks Spike protein entry. HeLa cells stably expressing ACE2 were transfected with LifeAct-CFP-FRB (blue) and ACE2-mNeon-FKBP (green). Transfected cells were treated with 0.1% DMSO or 100 nM rapamycin (Rapa) for 5 min and then incubated with SARS-CoV-2 spike-Alexa Fluor 647 (purple; 1.2 ng/μl) for 5 min. After washing out unbound Spike-Alexa Fluor 647, the internalization of Spike protein was imaged every 10 min for 2 h. Scale bar, 10 μm. See also Figure 7b.

Video S30
Dimerization occurs only in ACE2-FKBP-labeled components at plasma membrane. COS7 cells co-transfected with ACE2-mNeon-FKBP (green) and CFP-FRB (blue) were treated with 100 nM rapamycin (Rapa). Images were taken every 10 s for 480 s. Scale bar, 10 μm. See also Figure S18a, Supporting Information.

Video S31
RIVET rapidly induces dimerization between vesicles and cortical actin. COS7 cells co-transfected with LifeAct-CFP-FRB (green) and mNeon-FKBP (upper panel) and ACE2-mNeon-FKBP (lower panel), respectively, were treated with 100 nM rapamycin (Rapa). FRET based images were taken every 10 s for 240 s. Scale bar, 10 μm. See also Figure S19a, Supporting Information.

Supporting information of protein sequence

ACE2 used in *ACE2-mNeon-FKBP*

MSSSSWLLLSLVAVTAAQSTIEEQAKTFLDKFNHEAEDLFYQSSLASWNYNTNITEENVQNMNNAGDKWSAFLKEQSTLAQMYPLQEIQ
NLTVKLQLQALQQNGSSVLSEDKSKRLNTILNTMSTIYSTGKVCNPDNPQECLLLEPGLNEIMANSLDYNERLWAWESWRSEVGKQLRPL
YEEYVVLKNEMARANHYEDYGDYWRGDYEVNGVDGYDYSRGQLIEDVEHTFEEIKPLYEHLHAYVRAKLMNAYPSYISPIGCLPAHLLGD
MWGRFWTNLYSLTVPFGQKPNIDVTDAMVDQAWDAQRIFKEAEKFFVSVGLPNMTQGFWENSMLTDPGNVQKAVCHPTAWDLGKG
DFRILMCTKVTMDDFLTAHHEMGHIQYDMAYAAQPFLLRNGANEGFHEAVGEIMSLSAATPKHLKSIGLLSPDFQEDNETEINFLLKQALT
IVGTLPFTYMLEKWRWMVFKGEIPKDQWMKKWWEMKREIVGVVEPVPHDETYCDPASLFHVSNDYSFIRYYTRTLYQFQFQEALCQAA
KHEGPLHKCDISNSTEAGQKLFNMLRLGKSEPWTLALENVVGAKNMNVRPLLNYFEPLFTWLKDQNKNSFVGWSTDWSPYADQSIKVR
ISLKSALGDKAYEWNDNEMYLFRSSVAYAMRQYFLKVKNQMILFGEEDVRVANLKPRISFNFFVTAPKNVSDIIPRTEVEKAIRMSRSRIND
AFRLNDNSLEFLGIQPTLGPPNQPPVSIWLIVFGVVMGVIVVGIVILIFTGIRDRKKKNKARSGENPYASIDISKGENNPGFQNTDDVQTSF

LAMP1 used in *LAMP1-mNeon-FKBP and LAMP1-mCherry-Cry2*

MAAPGSARRPLLLLLLLLLLGLMHCASAAMFMVKNGNGTACIMANFSAAFSVNYDTKSGPKNMTFDLPSDATVVLNRSSCGKENTSDPS
LVIAFGRGHTLTLNFTRNATRYSVQLMSFAYNLSDTHLFPNASSKEIKTVESITDIRADIDKKYRCVSGTQVHMNNVTVTPHDATIQAYLSNS
SFSRGETRCEQDRPSPTTAPPAPPSPSPSPVPKSPSVDKYNVSGTNGTCLLASMGLQLNLTYERKDNTTVTRLLNINPNKTSASGSCGAHLV
TLELHSEGTTVLLFQFGMNASSSRFFLQGIQLNTILPDARDPAFKAANGSLRALQATVGNSYKCNAEEHVRVTKAFSVNIFKVWVQAFKVE
GGQFGSVEECLLDENSMLIPIAVGGALAGLVLIVLIAYLVGRKRSHAGYQTI

LAMP3 used in *LAMP3-mNeon-GAIs*

MAVEGGMKCVKFLLYVLLLAFCACAVGLIAVGVGAQLVLSQTIIQGATPGSLLPVVIIAVGVFLFLVAFVGCCGACKENYCLMITFAIFLSLIML
VEVAAAIAGYVFRDKVMSEFNNNFRQQMENYPKNNHTASILDRMQADFKCCGAANYTDWEKIPSMSKNRVPDSCCINVTVGCGINFN
EKAIHKEGCVEKIGGWLRKNVLVVAAAALGIAFVEVLGIVFACCLVKSIRSGYEVM

PCM1F2 used in *PCM1F2-mNeon-FKBP*

MQDDDPEPQVLTANASNMGDFLGEMEETKQQPNNVRVSTNKLQKDAGLNEKAREKFYEAKLQQQQRELKQLQEERRKLMEIQEKIEVL
QKACPDLQSAGLGNSPANRQTSPATSTPAMNECNTAGKPLLEFGESVPVGNELWSEMRRHEILREELRRRRKQLEALMAEHQRRRELAET
ISTVAASVKSEGSEAQRTPQQSRTENRTMATWGGSTQCALDEEDGDEDGYLSDGLDQAEEEEDAPSMNDSFSAYPNNQIPESVYYLKGN
KDRWKNCRPLSADGNYRPMSKTRQQQNISMRRQENFRWISXLSYVEEKEQWQEQINQLKKQLEFSVSICQTLMQDQQTLSCFLQTLLA
GPYNVVPNNVASSQVHLIMHQLNQCYTQLSWQQNNVQRLKQMLNDLMHQQEQQCQEKPSRKERGSSAPPPPSPVFCPFSFPPQPVN
LFNIPGFTNISSFAPGINYNPVFPCGFGDFAHSGFPQSSEQQQHPLDHNASGKTEYMAFPKPFESSSSTGAENQRSHRQPEDEVEKRSTW
LNDSQEVKKDDQSQQKAGFPVSVQSIASGHKNQSDTSRRRNFDEESLESFSSMPDPVDPTTVTKTFKSRKASAQASLASKDKTPKSKNKR
KNSSQLKGRIKNTGYDSASASSVCEPCKSTKSKHSEEVVHAKVFSKKNREQLEKIIKYSRSTEMSSETGSDLSMFEALRDTIYSEVATLISQNES
RPHFLIELFHELQLLNTDYLRQRALYALQDIVTRHLSENNEKGRCIKSLNTATWIASNSELTPSESLASTDDETFDKNFPTEACQDCEQNDAD
NGSTMSTSSHFEPFATDDLGNTVIHLDQALARMREYERMKIEAESTLDSEGCSSNLQGATAAK

Pex used in *Pex-mCherry and Pex-mNeon-FKBP*

MLRSVWNFLKRHKKKCIFLGTVLGGVYILGKYGQKKIREIQER

TGN38 used in *TGN38-mCherry, TGN38-mNeon-FKBP, and TGN38-mNeon-GAIs*

MRFVVALVLLNVAAAGAVPLLATESVKQEEAGVRPSAGNVSTHPSLSQRPGGSTKSHPEPQTPKDSPSKSSAEAQTPEDTPNKSGAEAKT
QKDSSNKSGAEAKTQKGSTSKSGSEAQTTKDSTSKSHPELQTPKDSTGKSGAEAQTPEDSPNRSGAEAKTQKDSPSKSGSEAQTTKDVPN
KSGADGQTPKDGSSKSGAEDQTPKDVPNKSGAEKQTPKDGSNKSGAEEQGPIDGPSKSGAEEQTSKDSPNKVVPEQPSRKDHSKPISNP
SDNKELPKADTNQLADKGKLSPHAFKTESGEETDLISPPQEEVKSSEPTEDVEPKEAEDDDTGPEEGSPPKEEKEKMSGSASSENREGTLSD
STGSEKDDLYPNGSGNGSAESSHFFAYLVTAAILVAVLYIAHHNKRKIIAFVLEGKRSKVTRRPKASDYQRLDQK

Rab5b used in *mNeon-FKBP-Rab5b and mCherry-FKBP-Rab5b*

MTSRSTARPNGQPQASKICQFKLVLLGESAVGKSSLVLRFVKGQFHEYQESTIGAAFLTQSVCLDDTTVKFEIWDTAGLERYHSLAPMYYRG
AQAAIVVYDITNQETFARAKTWVKELQRQASPSIVIALAGNKADLANKRMVEYEEAQAYADDNSLLFMETSAKTAMNVNDLFLAIAKKLP
KSEPQNLGGAAGRSRGVDLHEQSQQNKSQCCSN

Rab11a used in *mNeon-FKBP-Rab11a*

MYELYKSGLRSMGTRDDEYDYLFKVVLIGDSGVGKSNLLSRFTRNEFNLESKSTIGVEFATRSIQVDGKTIKAQIWDTAGLERYRAITSAYYR
GAVGALLVYDIAKHLTYENVERWLKELRDHADSNIVIMLVGNKSDLRHLRAVPTDEARAFAEKNGLSFIETSALDSTNVEAAFQTILTEIYRIV
SQKQMSDRRENDMSPSNNVVPIHVPPTTENKPKVQCCQNI

Rab11b used in *mNeon-FKBP-Rab11b*

MGTRDDEYDYLFKVVLIGDSGVGKSNLLSRFTRNEFNLESKSTIGVEFATRSIQVDGKTIKAQIWDTAGLERYRAITSAYYRGAVGALLVYDIA
KHLTYENVERWLKELRDHADSNIVIMLVGNKSDLRHLRAVPTDEARAFAEKNNLSFIETSALDSTNVEEAFKNILTEIYRIVSQKQIADRAAH
DESPGNNVVDISVPPTTDGQKPNKLQCCQNL

Rab37 used in *mNeon-FKBP-Rab37*

MTGTPGAVATRDGEAPERSPPCSPSYDLTGKVMLLGDTGVGKTCFLIQFKDGAFLSGTFIATVGIDFRNKVVTVDGVRVKLQIWDTAGLER
FRSVTHAYYRDAQALLLLYDITNKSSFDNIRAWLTEIHEYAQRDVVIMLLGNKADMSSERVIRSEDGETLAREYGVPFLETSAKTGMNVELA
FLAIAKELKYRAGHQADEPSFQIRDYVESQKKRSSCCSFM

VAMP2 used in *mNeon-FKBP-VAMP2 and mCherry-Cry2-VAMP2*

MSATAATVPPAAPAGEGGPPAPPPNLTSNRRLQQTQAQVDEVVDIMRVNVDKVLERDQKLSELDDRADALQAGASQFETSAAKLKRKY
WWKNLKMMIILGVICAIILIIIIVYFST

IFT88 used in *mNeon-IFT88 and mNeon-FKBP-IFT88*

MMENVHLAPETDEDDLYSGFNDYNPAYDTEELENDTGFQQAVRTSHGRRPPVTAKIPSTAVSRPIATGYGSKTSLTSSMGRPMTGTIQDG
VARPMTAVRAAGFSKAALRGSAFDPLGQSRGPAPPLEAKNEDSPEEKIRQLEKKVNELVEESCIANSCGDLKLALEKAKDAGRKERVLVRQ
REQVTSPENINLDLTYSVLFNLASQYSANEMYAEALNTYQVIVKNKMFSNAGRLKVNMGNIYLKQRNYSKAIKFYRMALDQIPSVHKEMRI
KIMQNIGITFIKTGQYSDAINSFEHIMSMAPSLKAGFNLILSCFAIGDREKMKKAFQKLIAVPLEIDEDDKYISPSDDPHTNLLIEAIKNDHLRQ
MERERKAMAEKYIMTAAKLIAPVIEASFAVGYNWCVEVVKASQYVELANDLEINKAITYLRQKDFNQAVDTLKMFEKKDSRVKSAAATNL
SFLYYLENEFAQASSYADLAVNSDRYNPSALTNKGNTVFANGDYEKAAEFYKEALRNDSSCTEALYNIGLTYKKLNRLDEALDSFLKLHAILRN
SAQVLCQIANIYELMEDPNQAIEWLMQLISVVPTDSQALSKLGELYDSEGDKSQAFQYYYESYRYFPSNIEVIEWLGAYYIDTQFCEKAIQYF
ERASLIQPTQVKWQLMVASCFRRSGNYQKALDTYKEIHRKFPENVECLRFLVRLCTDIGLKEVQEYATKLKRLEKMKEMREQRIKSGRDSSG
GSRSKREGSAGSDASYVDPLGPQIERPKTAAKKRIDEDDFADEELGDDLLPE

Rab7a used in *RFP-Rab7a*

MNSRRACDRDPDSSCRRRVIYKVKLRGTNFPSDGPVMQKKTMGWEASTERMYPEDGALKGEIKMRLKLKDGGHYDAEVKTTYMAKKP
VQLPGAYKTDIKLDITSHNEDYTIVEQYERAEGRHSTGALYKYSDLEMTSRKKVLLKVIILGDSGVGKTSLMNQYVNKKFSNQYKATIGADFL
TKEVMVDDRLVTMQIWDTAGQERFQSLGVAFYRGADCCVLVFDVTAPNTFKTLDSWRDEFLIQASPRDPENFPFVVLGNKIDLENRQVA
TKRAQAWCYSKNNIPYFETSAKEAINVEQAFQTIARNALKQETEVELYNEFPEPIKLDKNDRAKTSAESCSC

5HT6 used in *5HT6-mCherry*

MVPEPGPVNSSTPAWGPGPPPAPGGSGWVAAALCVVIVLTAAANSLLIALICTQPALRNTSNFFLVSLFTSDLMVGLVVMPPAMLNALYG
RWVLARGLCLLWTAFDVMCCSASILNLCLISLDRYLLILSPLRYKLRMTAPRALALILGAWSLAALASFLPLLLGWHELGKARTSAPGQCRLL
ASLPYVLVASGVTFFLPSGAICFTYCRILLAARKQAVQVASLTTGTATAGQALETLQVPRTPRPGMESADSRRLTTKHSRKALKASLTLGILLS
MFFVTWLPFFVASIAQAVCDCISPGLFDVLTWLGYCNSTMNPIIYPLFMRDFKRALGRFVPCVHCPPEHRASPASPSMWTSHSGARPGLS
LQQVLPLPLPPNSDSDSASGGTSGLQLTAQLLLPGEATRDPPPPTRAPTVVNFFVTDSVEPEIRQHPLGSPMN

EMTB used in *EMTB-CFP-FRB, EMTB-CFP-mGID1, EMTB-YFP-CIBN, and EMTB-RFPnano-Cry2*

MEQKLISEEDLGAPVRSETAPDSYKVQDKKNASSRPASAISGQNNNHSGNKPDPPPVLRVDDRQRLARERREEREKQLAAREIVWLEREE RARQHYEKHLEERKKRLEEQRQKEERRRAAVEEKRRQRLEEDKERHEAVVRRTMERSQKPKQKHNRWSWGGSLHGSPSIHSADPDRRS VSTMNLSKYVDPVISKRLSSSSATLLNSPDRARRLQLSPWESSVVNRLLTPTHSFLARSKSTAALSGEAASCSPIIMPYKAAHSRNSMDRPKL FVTPPEGSSDP

MAPTau used in *CFP-FRB-MAPTau and mNeon-MAPTau*

MAEPRQEFEVMEDHAGTYGLGDRKDQGGYTMHQDQEGDTDAGLKAEEAGIGDTPSLEDEAAGHVTQARMVSKSKDGTGSDDKKAK GADGKTKIATPRGAAPPGQKGQANATRIPAKTPPAPKTPPSSGEPPKSGDRSGYSSPGSPGTPGSRSRTPSLPTPPTREPKKVAVVRTPPKS PSSAKSRLQTAPVPMPDLKNVKSKIGSTENLKHQPGGGKVQIVYKPVDLSKVTSKCGSLGNIHHKPGGGQVEVKSEKLDFKDRVQSKIGSL DNITHVPGGGNKKIETHKLTFRENAKAKTDHGAEIVYKSPVVSGDTSPRHLSNVSSTGSIDMVDSPQLATLADEVSASLAKQGL

LifeAct used in *LifeAct-CFP-FRB*

MGVADLIKKFESISKEEG

FKBP used in *mNeon-FKBP, LAMP1-mNeon-FKBP, PCM1F2-mNeon-FKBP, Pex-mNeon-FKBP, Pex-mNeon-FKBP, TGN38-mNeon-FKBP, mNeon-FKBP-Rab5b, mNeon-FKBP-Rab11a, mNeon-FKBP-Rab11b, mNeon-FKBP-Rab37, and mNeon-FKBP-VAMP2*

GVQVETISPGDGRTFPKRGQTCVVHYTGMLEDGKKFDSSRDRNKPFKFMLGKQEVIRGWEEGVAQMSVGQRAKLTISPDYAYGATGHP GIIPPHATLVFDVELLKLE

FRB used in *EMTB-CFP-FRB, LifeAct-CFP-FRB, and CFP-FRB-MAP4m*

ILWHEMWHEGLEEASRLYFGERNVKGMFEVLEPLHAMMERGPQTLKETSFNQAYGRDLMEAQEWCRKYMKSGNVKDLLQAWDLYYH VFRRISK

GAIs used in *LAMP3-mNeon-GAIs and TGN38-mNeon-GAIs*

MKRDHHHHHHQDKKTMMMNEEDDGNGMDELLAVLGYKVRSSEMADVAQKLEQLEVMMSNVQEDDLSQLATETVHYNPAELYTWL DSMLTDLN

mGID1 used in *EMTB-CFP-mGID1*

MAASDEVNLIESRTVVPLNTWVLISNFKVAYNILRRPDGTFNRHLAEYLDRKVTANANPVDGVFSFDVLIDRRINLLSRVYRPAYADQEQPP SILDLEKPVDGDIVPVILFFHGGSFAHSSANSAIYDTLCRRLVGLCKCVVVSVNYRRAPENPYPCAYDDGWIALNWVNSRSWLKSKKDSKV HIFLAGDSSGGNIAHNVALRAGESGIDVLGNILLNPMFGGNERTESEKSLDGKYFVTVRDRDWYWKAFLPEGEDREHPACNPFSPRGKSL EGVSFPKSLVVVAGLDLIRDWQLAYAEGLKKAGQEVKLMHLEKATVGFYLLPNNNHFHNVMDEISAFVNAEC

Cry2 used in *LAMP1-mCherry-Cry2 and mCherry-Cry2-VAMP2*

MKMDKKTIVWFRRDLRIEDNPALAAAAHEGSVFPVFIWCPEEEGQFYPGRASRWWMKQSLAHLSQSLKALGSDLTLIKTHNTISAILDCI RVTGATKVVFNHLYDPVSLVRDHTVKEKLVERGISVQSYNGDLLYEPWEIYCEKGKPFTSFNSYWKKCLDMSIESVMLPPPWRLMPITAAA EAIWACSIEELGLENEAEKPSNALLTRAWSPGWSNADKLLNEFIEKQLIDYAKNSKKVVGNSTSLLSPYLHFGEISVRHVFQCARMKQIIWA RDKNSEGEESADLFLRGIGLREYSRYICFNFPFTHEQSLLSHLRFFPWDADVDKFKAWRQGRTGYPLVDAGMRELRATGWMHNRIRVIVS SFAVKFLLLPWKWGMKYFWDTLLDADLECDILGWQYISGSIPDGHELDRLDNPALQGAKYDPEGEYIRQWLPELARLPTEWIHHPWDAP LTVLKASGVELGTNYAKPIVDIDTARELLAKAISRTREAQIMIGAA

CIBN used in *EMTB-YFP-CIBN*

MNGAIGGDLLLNFPDMSVLERQRAHLKYLNPTFDSPLAGFFADSSMITGGEMDSYLSTAGLNLPMMYGETTVEGDSRLSISV

MAP4m used in *CFP-FRB-MAP4m*

MSGSKSTQTVAKTTTAAAVASTGPSSRSPSTLLPKKPTAIKTEGKPAEVKKMTAKSVPADLSRPKSTSTSSMKKTTTLSGTAPAAGVVPSRVK
ATPMPSRPSTTPFIDKKPTSAKPSSTTPRLSRLATNTSAPDLKNVRSKVGSTENIKHQPGGGRVQIVSKKVSYSHIQSKCGSKDNIKHVPGG
GNVQIQNKKVDISKVSSKCGSKANIKHKPGGGDVKIESQKLNFKEKAQAKVGSLDNVGHLPAGGAVKTEGGGSEAPLCPGPPAGEEPAISE
AAPEAGAPTSASGLNGHPTLSGGGDQREAQTLDSQIQETSI

Rab3 used in *mCh-CIBN-Rab3-SL2-EMTB-RFPnano-Cry2*

MNNQQAAIASARSRMAAGGQPQGATPGQPDQNFDYMFKLLIIGNSSVGKTSFLFRYCDDSFTSAFVSTVGIDFKVKTVFRGDKRVKLQI
WDTAGQERYRTITTAYYRGAMGFILMYDITNEESFNSVQDWCTQIKTYSWENAQVVLVGNKCDMDSERVVSMDRGRQLADQLGLEFFE
TSAKENINVKAVFEKLVEIICDKMAESLDKDPQQQPKGQKLEANPTQKPAQQQCNC
